# Global losses due to dairy cattle diseases: A comorbidity-adjusted economic analysis

**DOI:** 10.3168/jds.2023-24626

**Published:** 2024-09

**Authors:** Philip Rasmussen, Herman W. Barkema, Prince P. Osei, James Taylor, Alexandra P. Shaw, Beate Conrady, Gemma Chaters, Violeta Muñoz, David C. Hall, Ofosuhene O. Apenteng, Jonathan Rushton, Paul R. Torgerson

**Affiliations:** 1 Section of Animal Welfare and Disease Control, Department Veterinary and Animal Sciences, University of Copenhagen, Copenhagen DK-1870, Denmark; 2 Section of Epidemiology, Vetsuisse Faculty, University of Zurich, Zurich CH 0857, Switzerland; 3 Global Burden of Animal Diseases (GBADs), Liverpool L69 3BX, United Kingdom; 4 Faculty of Veterinary Medicine, University of Calgary, Calgary, AB T2N 4Z6, Canada; 5 School of Mathematics and Statistics, Carleton University, Ottawa, ON K1S 5B6, Canada; 6 Agri-Food and Biosciences Institute (AFBI), Belfast BT9 5PX, United Kingdom; 7 Department of Livestock and One Health, Institute of Infection, Veterinary & Ecological Sciences, University of Liverpool, Liverpool L69 3BX, United Kingdom; 8 Infection Medicine, Biomedical Sciences, Edinburgh Medical School, University of Edinburgh, Edinburgh EH16 4SB, United Kingdom

**Keywords:** dairy, disease, economic, impact

## Abstract

An economic simulation was carried out over 183 milk-producing countries to estimate the global economic impacts of 12 dairy cattle diseases and health conditions: mastitis (subclinical and clinical), lameness, paratuberculosis (Johne's disease), displaced abomasum, dystocia, metritis, milk fever, ovarian cysts, retained placenta, and ketosis (subclinical and clinical). Estimates of disease impacts on milk yield, fertility, and culling were collected from the literature, standardized, meta-analyzed using a variety of methods ranging from simple averaging to random-effects models, and adjusted for comorbidities to prevent overestimation. These comorbidity-adjusted disease impacts were then combined with a set of country-level estimates for lactational incidence or prevalence or both, herd characteristics, and price estimates within a series of Monte Carlo simulations that estimated and valued the economic losses due to these diseases. It was estimated that total annual global losses are US$65 billion (B). Subclinical ketosis, clinical mastitis, and subclinical mastitis were the costliest diseases modeled, resulting in mean annual global losses of approximately US$18B, US$13B, and US$9B, respectively. Estimated global annual losses due to clinical ketosis, displaced abomasum, dystocia, lameness, metritis, milk fever, ovarian cysts, paratuberculosis, and retained placenta were estimated to be US$0.2B, US$0.6B, US$0.6B, US$6B, US$5B, US$0.6B, US$4B, US$4B, and US$3B, respectively. Without adjustment for comorbidities, when statistical associations between diseases were disregarded, mean aggregate global losses would have been overestimated by 45%. Although annual losses were greatest in India (US$12B), the United States (US$8B), and China (US$5B), depending on the measure of losses used (losses as a percentage of gross domestic product, losses per capita, losses as a percentage of gross milk revenue), the relative economic burden of these dairy cattle diseases across countries varied markedly.

## Abstract (author highlights)

The list of standard abbreviations for JDS is available at adsa.org/jds-abbreviations-24. Nonstandard abbreviations are available in the Notes.

## INTRODUCTION

In 2021, global milk production approached one billion (**B**) metric tonnes, with over 80% of that production coming from cattle (FAOSTAT, 2023b). Milk is as an important source of nutrients, and nutrient-rich foods such as milk are anticipated to continue playing a key role in global nutrition and food security (Smith et al., 2022), with total global food demand expected to increase by up to 56% between 2010 and 2050 (van Dijk et al., 2021). The consumption of dairy products by humans is associated with improved bone mass bone mass (McCabe et al., 2004), cardiovascular health (Zemel, 2004), and gastrointestinal health (Gorbach, 2000), and milk is an important source of nutrition for infants and children, who require nutrient- and energy-rich foods for growth and cognitive development (Garcia et al., 2019). Dairy cattle are also an integral part of the global economy. For example, in the European Union, milk is the second most produced food product after fruits and vegetables and accounts for approximately 14% of agricultural production (Yilmaz, 2017). Cattle and other livestock also often serve as a form of wealth storage, particularly in lower-income and less developed countries (Tabe Ojong et al., 2022).

Dairy cattle and other livestock also play an important role in upcycling less-edible material, as well as co-products and byproducts of other agricultural production processes, into milk and dairy products (Peterson and Mitloehner, 2021). However, the industry is associated with high levels of air and water pollution in the form of greenhouse gases, such as methane, that are produced through fermentation, as well as nitrogen emissions through feces and urine (Peterson and Mitloehner, 2021). Dairy cattle diseases and health conditions that negatively affect cow productivity exacerbate this issue by reducing the efficiency of milk production. Therefore, it is necessary to better understand the global economic losses due to dairy cattle diseases and explore how these losses are distributed across diseases and populations. Accordingly, this economic analysis aims to estimate the global losses due to 12 dairy cattle diseases across 183 milk-producing countries to guide the formulation of effective, evidence-based animal health policy at the farm, national, and global levels.

When estimating the economic losses due to multiple diseases, it is important to consider that animals may have concurrent diseases and conditions, or comorbidities, particularly if the economic model will be at the average animal level (e.g., a snapshot that is representative of the average state of an animal). If impact estimates across multiple diseases are combined and comorbidities are not considered, there is the potential to double count impacts and, therefore, overestimate losses (Rasmussen et al., 2022b). This potential for double counting and the resulting overestimation has been explored from both the human and animal health perspectives. For example, Olesen et al. (2012) estimated the economic costs of a variety of brain diseases in Europe but observed that a substantial proportion of patients had multiple diagnoses (i.e., depression and anxiety disorders) and reduced the aggregate number of patients in the economic analysis to mitigate the risk of double counting. McDonald et al. (2020) recognized that the occurrence of 2 or more medical conditions in a single individual is common, and that if disability-adjusted life year calculations were to be carried out for each condition separately, comorbidities could lead to overestimation.

From the animal health perspective, this potential for overestimation is discussed in Torgerson and Shaw (2021), but few studies explicitly account for statistical associations between diseases in their economic analyses. For example, in an effort to avoid double counting and overestimating the economic value of an array of genetic traits, Østergaard et al. (2016) introduced mediator variables when simulating the economic values of cattle breeding goals. When modeling the economic impact of subclinical ketosis (**SCK**) in dairy cattle, Raboisson et al. (2015) reported that the impact of the disease would be overestimated by 68% if raw impact estimates from the literature were used without adjustment for associations between SCK and various other cattle diseases and health conditions. Rasmussen et al. (2022b) describes a framework using Bayes' Theorem to adjust disease impact estimates for comorbidities before economic analysis using disease probabilities, disease impacts, and disease association estimates. As part of the Global Burden of Animal Diseases (**GBADs**) program, an international collaboration aiming to measure and improve societal outcomes from livestock (Rushton et al., 2018; Huntington et al., 2021), the framework described by Rasmussen et al. (2022b) was used to estimate the global comorbidity-adjusted economic losses due to an array of dairy cattle diseases and health conditions. This model is being considered as a critical component of the overall GBADs program to assess animal diseases at a global level.

## MATERIALS AND METHODS

This economic simulation considered the global impacts on milk production, fertility, and culling of 12 cattle diseases and health conditions: mastitis (subclinical mastitis [**SCM**] and clinical mastitis [**CM**]), lameness (**LAM**), paratuberculosis (**PTB**; Johne's disease), displaced abomasum (**DA**), dystocia (**DYS**), metritis (**MET**), milk fever (**MF**), ovarian cysts (**OC**), retained placenta (**RP**), and ketosis (SCK and clinical ketosis [**CK**]). Estimates of disease impacts on productivity were collected from the literature, standardized, meta-analyzed using a variety of methods ranging from simple averaging to random-effects models, and adjusted for comorbidities to prevent overestimation. These comorbidity-adjusted impacts were then combined with a set of country-level lactational incidence or prevalence (depending on the characteristics of the disease or health condition estimates), herd characteristics, and price estimates within a series of Monte Carlo simulations that estimated and valued the economic losses due to these diseases. Forgone milk yield was valued using the price of milk; increased calving interval was valued using the number of days calving was delayed, daily milk production, and the price of milk; and increased risk of premature culling was valued using the price of replacement cows and heifers less the sale price of culled cows.

### Production Parameters

**Figure 1:**
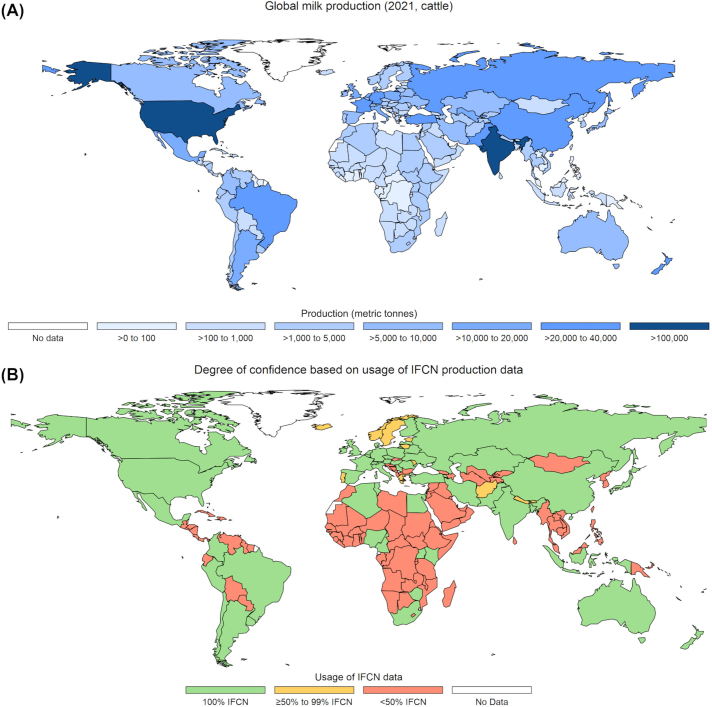
Global milk production and the availability of farm production data. (A) Global milk production (metric tonnes of cow's milk) in 2021 (FAOSTAT, 2023b). (B) Availability of International Farm Comparison Network data (IFCN, 2023) and the resulting degree of confidence in the production values used as inputs in the estimation of economic losses.

Herd production parameters were obtained through a confidentiality agreement with the International Farm Comparison Network (**IFCN**), which provided a data set of key productivity measures and herd characteristics for the year 2021 based on typical dairy farms in 53 countries (IFCN, 2023). This data set captured countries accounting for over 80% of global dairy production in 2021 (Figure 1). Due to the stipulations of the confidentiality agreement, all IFCN data must be reported only at the regional (i.e., continental regions) and global (i.e., globally aggregated) levels, and all results of this study must be reported such that the extraction of country-level IFCN data is prevented.

Although over 80% of global milk production was captured by the 53 countries in the IFCN data set, an additional 130 countries had produced cow's milk in 2021, according to the records of the Food and Agriculture Organization of the United Nations (**FAO**; FAOSTAT, 2023b). For these 130 countries, which accounted for less than 20% of global dairy production in 2021, farm characteristics were approximated based on the values provided by IFCN for geoeconomically similar countries. Thus, a national stratification structure that incorporated not only geographic but economic characteristics was required, and accordingly, countries were grouped using the Global Burden of Diseases project structure (GBD, 2023). This resulted in 21 subregions used for country-level approximations: Southeast Asia, Oceania, East Asia, South Asia, North Africa and the Middle East, 4 subregions within Sub-Saharan Africa (Western, Southern, Central, and Eastern), 4 subregions within Latin America and the Caribbean (Tropical, Caribbean, Andean, and Central), 3 subregions within Europe and Central Asia (Central Asia, Central Europe, and Eastern Europe), and 5 subregions within the High Income category (Western Europe, Southern Latin America, North America, Asia Pacific, and Australasia).

Countries for which approximation was required were then ranked according to the percentage of countries within their subregion for which IFCN data were available to reflect the accuracy of the values used in this simulation and, therefore, the confidence with which the resulting economic estimates should be interpreted (Figure 1B). Additional data on the production (FAOSTAT, 2023b) and price (FAOSTAT, 2023c) of milk were obtained from the FAO and used to estimate the national herd (annual national production divided by average annual production per cow) and the average daily milk yield (annual production per cow divided by 365.25 d/yr) for all 183 countries with FAO milk production records in 2021. All production parameters are summarized at the continental region level in Table 1.

**Table 1:** Regional summary of country-level input values used in the estimation of global economic losses due to dairy cattle diseases^1^

Item	Africa	Asia	Europe	Latin America	North America	Oceania	Global	Source
Price of milk (US$/100 kg)	72.42	64.72	41.55	53.31	45.85	79.69	59.81	FAOSTAT (2023c)
National milk production (millions of metric tonnes/yr)	0.82	5.55	5.63	2.27	41.65	2.80	4.08	FAOSTAT (2023b)
Annual milk yield (kg/cow)	2,533.92	4,596.59	7,436.83	5,463.73	8,901.09	5,965.25	5,013.36	Adapted from IFCN data^2^
National herd (million of cows)	0.30	2.54	0.78	0.42	4.38	0.49	1.01	Calculated^3^
Calving interval (d)	455.04	428.29	406.20	434.60	408.33	365.00	427.43	Adapted from IFCN data^2^
Cow replacement rate (%)	20.55	20.43	29.58	22.67	32.25	26.50	23.66	Adapted from IFCN data^2^
Replacement price (US$/cow)	812.44	1,899.38	1,538.99	1,070.08	1,521.07	1,044.95	1,299.33	Adapted from IFCN data^2^, ^4^
Culled cow price (US$/cow)	599.37	1,235.49	892.96	480.12	779.32	485.51	785.86	Adapted from IFCN data^2^

1 Although country-level data were used in the analyses, due to a confidentiality agreement, data from the International Farm Comparison Network (IFCN) can only be reported at the regional level. All other data are open access and available at the country level from their sources, as described in the table.

2 Country-level data from the IFCN were used to approximate values in countries without IFCN data according to socioeconomic regions defined by the Global Burden of Disease project (GBD, 2023).

3 National milk production divided by annual milk yield per cow.

4 Price for a replacement heifer or cow.

### Diseases

The analyses included 12 commonly studied and reported diseases: DA, DYS, SCK, CK, LAM, SCM, CM, MET, MF, OC, PTB, and RP.

Displaced abomasum is a common disorder of high-producing dairy cattle (Wittek, 2022) characterized by the displacement of the abomasum from its normal position to the right or left side in cattle, with left abomasal displacement being more frequently diagnosed than right (LeBlanc et al., 2005; Caixeta et al., 2018). Displaced abomasum is a multifactorial disorder diagnosed almost exclusively in adult dairy cows (Caixeta et al., 2018), with clinical signs including anorexia and decreased milk production (Detilleux et al., 1997; Raizman and Santos, 2002), and cows diagnosed with DA having a higher risk of culling (Geishauser et al., 1998; Gröhn et al., 1998, Raizman and Santos, 2002).

Dystocia, or the difficulty or inability of a dam to deliver its young through its own effort, can result in calf loss (Abera, 2017) and is a common problem among dairy cows (Tenhagen et al., 2007). Calf birth weight, malpresentation, congenital deformities, and the size of the pelvic area of the dam are some of the determinants of DYS, with severe cases requiring veterinary intervention (Statham, 2023). Dystocia can result in reduced milk production (Mangurkar et al., 1984; Djemali et al., 1987; Simerl et al., 1992; Dematawewa and Berger, 1997; Rajala and Gröhn, 1998), reduced fertility (Fourichon et al., 2000), and an increased risk of culling (Rajala-Schultz and Gröhn, 1999).

Ketosis, loosely defined as an elevated concentration of ketone bodies in the body's fluids, is a metabolic disorder affecting dairy cows and is associated with losses in milk production (Duffield, 2000; Raboisson et al., 2014), a prolonged calving interval (Fourichon et al., 2000; Mostert et al., 2018), and increased risk of periparturient disease (Duffield, 2000, Raboisson et al., 2014). Subclinical ketosis can be defined as high serum ketone concentrations without observed clinical signs (Duffield, 2022), and the prevalence of SCK in Europe has been estimated to be 25% (Raboisson et al., 2015). When cows with elevated concentrations of ketone bodies show clinical signs, the condition can be defined as CK (Steeneveld et al., 2020).

Lameness, or an abnormal gait resulting from injury, disease, or dysfunction of one or more feet or limbs (Whay and Shearer, 2017), is a prominent issue in the dairy industry (Dolecheck and Bewley, 2018) and is among the top health concerns for producers (Leach et al., 2010) and veterinarians (Bauman et al., 2016). Cows with severe forms of LAM suffer (Whay and Shearer, 2017), making it a significant problem for the industry from an animal welfare perspective (Whay et al., 2003). Lame animals typically spend more time lying on the floor and are therefore more likely to develop skin lesions and udder disorders, such as mastitis (Ózsvári, 2017). Lameness is associated with reduced milk production (Rowlands and Lucey, 1986; Tranter and Morris, 1991; Green et al., 2002), reduced fertility, (Fourichon et al., 2000), and an increased risk of culling (Rajala-Schultz and Gröhn, 1999; Sharifi et al., 2013).

Mastitis typically occurs when microbes enter the teat via the teat canal, with a marked inflammatory response to the invading pathogen signaling a progression from SCM to CM (Erskine, 2022). Both SCM and CM are associated with reduced milk production (Heikkilä et al., 2018), reduced reproductive performance (Fourichon et al., 2000; Klaas et al., 2004; Boujenane et al., 2015), and an increased risk of culling (Beaudeau et al., 1995; Rajala-Schultz and Gröhn, 1999; Hertl et al., 2011; Sharifi et al., 2013).

Metritis is used as a general term for postpartum uterine inflammation as a result of infection, which is common among cows and often occurs within the first 2 weeks after parturition (Lima, 2022). Infectious reproductive system diseases (e.g., brucellosis, leptospirosis, trichomoniasis, and campylobacteriosis) may also cause MET (Lima, 2022), and the disease is associated with substantial production losses (Rajala and Gröhn, 1998; Rajala-Schultz and Gröhn, 1999; Fourichon et al., 2000; Reppert, 2015).

Milk fever, postparturient hypocalcemia, or parturient paresis, is a metabolic disease occurring at the onset of lactation (Horst et al., 1997) characterized by low total serum calcium and inorganic phosphorus (Jorgensen, 1974). The field incidence of the disease generally ranges from 0% to 10%, but may exceed 25% of cows calving (DeGaris and Lean, 2008). Milk fever can be considered a gateway disease, greatly reducing the chance of full productivity throughout the subsequent lactation (Goff, 2008), and it has been associated with increased odds of other dairy cattle diseases, such as CK (Gröhn et al., 1989), MET, DYS (Gröhn et al., 1990), and LAM (Dohoo and Martin, 1984).

Ovarian cysts are an ovarian dysfunction whose definition and nomenclature has evolved over time (Borş and Borş, 2020). Formerly defined as follicular structures of at least 2.5 cm in diameter that persist for at least 10 d in the absence of a corpus luteum (Kesler and Garverick, 1982), the condition has since had a variety of names and accompanying clinical definitions (Garverick, 1997; Silvia et al., 2002; Vanholder et al., 2006; Youngquist and Threlfall, 2006). In contrast to rigid definitions previously used, this economic analysis will instead define the condition similarly to Borş and Borş (2020), who described it as an ovarian disorder characterized by abnormal ovarian cavity structures failing to ovulate or regress. Therefore, this study will loosely combine impact estimates across a variety of cystic ovarian disorders under the term OC. In general, the condition, as defined herein, is associated with reduced milk yield (Erb et al., 1985, Bigras-Poulin et al., 1990), reduced fertility (Klaas et al., 2004; Toni et al., 2015), and an increased risk of culling (Sharifi et al., 2013).

Paratuberculosis, or Johne's disease, is an infectious inflammatory disorder of the intestines affecting ruminants including dairy cattle (Fecteau and Whitlock, 2010). It is caused by an infection with *Mycobacterium avium* ssp. *paratuberculosis* (**MAP**), and as the disease progresses, its clinical effects worsen from diarrhea and reduced milk production to lethargy, hypoproteinemia, and severe emaciation (Tiwari et al., 2006). Paratuberculosis is associated with decreased milk production (Lombard et al., 2005; McAloon et al., 2016), reduced fertility (Johnson-Ifearulundu et al., 2000; Ózsvari et al., 2020), and premature culling (Ott et al., 1999; Shephard et al., 2016).

Retained placenta, or retention of fetal membranes, is a common postpartum disorder in cattle (Eppe et al., 2021) typically defined as a failure to expel fetal membranes within 24 h after calving (Roberts, 2022). Although recent studies suggest that RP is a multifactorial health issue involving aspects of the immune system, gene expression, and protein and metabolite alterations, the causes of RP remain uncertain (Dervishi and Ametaj, 2017). Risk factors for RP include abortion, DYS, twin birth, stillbirth, hypocalcemia, high environmental temperature, advancing age of the cow, premature birth or induction of parturition, placentitis, and nutritional disturbances (Roberts, 2022). The condition can result in significant economic losses (Laven and Peters, 1996; Dubuc et al., 2011) due to reduced milk yield (Rajala and Gröhn, 1998), reduced fertility (Fourichon et al., 2000), and increased risk of culling (Rajala-Schultz and Gröhn, 1999; Dubuc et al., 2011).

### Literature Search

The Advanced Search tool within the Scopus database (https://www.elsevier.com/products/scopus) was used to capture disease incidence, prevalence, or both; disease impact; and disease association estimates from the literature. For incidence and prevalence estimates, meta-analyses or multicountry studies were prioritized, in that order, and identified using the following search terms: “TITLE-ABS-KEY (“disease” AND “dairy” AND (“incidence” OR “prevalence”)). Similarly, for disease impacts, meta-analyses were prioritized, and whenever possible, reanalyzed with the inclusion of subsequent estimates. The following terms were used: “TITLE-ABS-KEY (“disease” AND “dairy” AND (“milk” OR “yield” OR “productivity” OR “culling” OR “reproduction” OR “fertility”) AND (“impact” OR “effect”)).” Finally, for estimates of statistical associations between diseases, the following terms were used: “TITLE-ABS-KEY ((“association” OR “odds ratio” OR “relationship” OR “effect” OR “link”) AND “dairy” AND “disease” AND “disease”).”

For the incidence and prevalence and disease impact searches, the “disease” term was replaced with the name of the disease being searched for, including any alternative names (e.g., “milk fever” OR “postparturient hypocalcemia” OR “parturient paresis”). For the disease association searches, within each pairwise search, the “disease” terms were replaced with a disease pair among the C(12, 2) = 66 (i.e. 12 choose 2) possible pairs given the 12 diseases being modeled. All searches were expanded to include relevant bibliographical entries from identified studies, and all searches were restricted to studies published within the period of January 1, 2000, to December 15, 2023. This period was only relaxed to include older publications if limited available estimates were available from within the defined period. In total, 341 relevant estimates were obtained and used in the analyses from a total of 4,636 screened publications. The incidence and prevalence estimates used as input values in the analyses are presented in Table 2 and Supplemental File S1 (see Notes), the pooled disease association estimates are presented in Table 3 and Supplemental File S2 (see Notes), and the disease impact estimates, before comorbidity adjustment, are presented in Table 4 and Supplemental Files S3 and S4 (see Notes)

**Table 2:** Estimated lactational incidence or prevalence (see footnote 1) of dairy cattle diseases used as input values in the comorbidity adjustment of dairy cattle disease impacts and the estimation of global economic losses (estimated mean and distribution parameters; global values presented are the average of national values weighted by national herd size, see Table 1)

Disease^1^	Distribution	Africa	Asia	Europe	Latin America	North America	Oceania	Global	Source(s)
CK	Beta	3.06 (shape1 = 13.95, shape2 = 441.59)							Suthar et al. (2013)^2^, ^3^
CM	Beta	18.70	29.33	45.32	14.83	37.26	12.90	30.49	Kossaibati et al. (1998), Krishnamoorthy et al. (2021)^4^, ^5^
	Shape1	52.71	8.13	7.25	4.46	3.53	2.10	8.55	
	Shape2	228.99	19.58	8.75	25.62	5.93	14.16	18.20	
DA	Beta	2.16 (shape1 = 27.50, shape 2 = 1,245.11)							Suthar et al. (2013)^2^, ^3^
DYS	PERT	5.99 (min = 1.90, max = 10.80)							Stevenson and Call (1988), Fourichon et al. (2001), Steinbock (2006), Atashi et al. (2012a)^6^
LAM	Beta	24.68	25.54	23.79	32.35	26.51	16.84	25.45	Adapted from Thomsen et al. (2023)^7^
	Shape1	145.33	7.15	71.96	11.24	45.83	18.26	78.29	
	Shape2	443.56	20.81	230.73	23.53	127.01	90.07	227.42	
MET	Beta	9.57 (shape1 = 17.73, shape2 = 167.35)							Gröhn et al. (1995), Suthar et al. (2013)^2^, ^3^, ^8^
MF	Beta	2.41 (shape1 = 7.76, shape2 = 313.41)							Gröhn et al. (1995), Suthar et al. (2013)^2^, ^3^, ^8^
OC	PERT	11.46 (min = 2.70, max = 19.07)							Stevenson and Call (1988), Borsberry and Dobson (1989), Mujuni et al. (1993), Melendez et al. (2003), Cattaneo et al. (2014)^9^
PTB	PERT	10.01 (min = 1.19, max = 21.08)							Adapted from Nielsen and Toft (2009)^10^
RP	Beta	12.35 (shape1 = 33.75, shape2 = 239.46)							Gröhn et al. (1995), Suthar et al. (2013)^2^, ^3^, ^8^
SCK	Beta	49.96	48.24	44.07	45.87	54.52	49.09	47.89	Duffield et al. (1998), Oetzel (2004), Oetzel (2013), Loiklung et al. (2022)^11^
	Shape1	411.74	21.84	67.67	4.58	131.50	11.52	178.14	
	Shape2	412.73	23.42	85.85	5.40	109.69	11.93	193.73	
SCM	Beta	44.03	42.04	37.25	35.60	46.11	36.95	40.97	Krishnamoorthy et al. (2021)^5^
	Shape1	204.27	156.98	40.23	7.71	39.42	11.61	116.23	
	Shape2	259.70	216.41	67.79	14.02	46.10	19.86	167.02	

1 Diseases are listed in alphabetical order. For SCM, due to an inability to identify lactational incidence estimates or case duration estimates in the literature, it was assumed that lactational incidence is approximately equivalent to prevalence. For PTB, a prevalence estimate was used due to the disease's chronic, lifelong nature. Therefore, for all diseases aside from PTB, the values reported are estimated lactational incidence rates in cases per 100 lactations. For PTB, the value reported is a cow-level percentage prevalence. CK = clinical ketosis; CM = clinical mastitis; DA = displaced abomasum; DYS = dystocia; LAM = lameness; MET = metritis; MF = milk fever; PTB = paratuberculosis; RP = retained placenta; OC = ovarian cyst; SCK = subclinical ketosis; SCM = subclinical mastitis; min = minimum; max = maximum. Shape1 and shape2 are the distribution parameters for the beta distribution (i.e., α and β).

2 Incidence (percentage) in a convenience sample of 593 dairy herds from 10 European countries from May to October 2011 converted to a proportion.

3 Meta-analysis of logit-transformed proportions with a generalized linear mixed model using the *metaprop* function from the *meta* R package (Balduzzi et al., 2019). Random-effects estimate. Details in Supplemental File S1 (see Notes).

4 Pooled continent-level prevalence estimates from Krishnamoorthy et al. (2021) were converted to incidence using an incidence-prevalence ratio of 39.9/25.9 = 1.5405. The incidence-prevalence ratio is based on a reported incidence of 39.9 cow-cases per 100 cows and 25.9% of cows being affected in a survey of 144 English herds between 1994 and 1996 (Kossaibati et al., 1998).

5 Beta distribution parameters approximated using a best-guess estimate (the mean value reported in the study) and an uncertainty range (the 95% CI reported in the study) using minimization, as described by Branscum et al. (2005), using the *betaExpert* function in the *prevalence* R package (Devleesschauwer et al., 2022).

6 Value from Stevenson and Call (1988) is the unweighted mean incidence. Value from Fourichon et al. (2001) is the reported incidence per 100 calving events. Values from Steinbock (2006) are the incidence (percentage) across Swedish Red and White and Holsteins in parities 1 and 2. Average across studies. Minimum and maximum values are the range of estimates.

7 Pooled continent-level prevalence estimates were adapted from Thomsen et al. (2023) using the *metamean* function in the *meta* R package (Baldizzi et al., 2019) with untransformed means. Random-effects pooled prevalence estimates were then converted to incidence using an incidence-prevalence ratio of 30.9/29.5 = 1.05. The incidence-prevalence ratio is based on a pooled estimated all-cause incidence of 30.9 cases per 100 cow-years and pooled a pooled prevalence of 29.5% (Afonso et al., 2020). Meta-analyses were stratified by region. Asia: Chapinal et al. (2014) and Sajid et al. (2021). Australia: Bonfatti et al. (2020) and Ranjbar et al. (2020). Europe: Clarkson et al. (1996), (Manske et al., 2002), Amory et al. (2006), Dembele et al. (2006), Dippel et al. (2009), Rouha-Mülleder et al. (2009), Rutherford et al. (2009), Barker et al. (2010), Šárová et al. (2011), Sarjokari et al. (2013), Griffiths et al. (2018), Sjöström et al. (2018), Randall et al. (2019), Browne et al. (2022), Jensen et al. (2022). North America: Espejo et al. (2006), Chapinal et al. (2013), King et al. (2016), Westin et al. (2016), Adams et al. (2017), Jewell et al. (2019), Denis-Robichaud et al. (2020), van Huyssteen et al. (2020), Warner et al. (2020), Matson et al. (2022). South America: Bran et al. (2018), Costa et al. (2018), Moreira et al. (2018). The estimate presented in the table for Africa is based on all estimates in all regions. Details in Supplemental File S1.

8 Value from Gröhn et al. (1995) is the lactation incidence risk.

9 Value from Stevenson and Call (1988) is the unweighted mean incidence. Value from Melendez et al. (2003) is the weighted mean incidence (percentage) across lame and non-lame cows.

10 Adapted from a literature review by Nielsen and Toft (2009), which generated animal-level true prevalence estimates. Estimates from studies without critical issues (Atala and Akcay, 2001; Petit, 2001; Robbi et al., 2002; Donat et al., 2005), as identified by the authors of the review, were averaged herein and assumed to be representative of the prevalence within infected herds. This mean within-infected-herd prevalence was then multiplied by the mean of apparent prevalence estimates at the herd-level, once again excluding studies with critical issues (Vicenzoni et al., 1999; Atala and Akcay, 2001; Petit, 2001; Robbi et al., 2002), as no herd-level true prevalence estimates were generated by the reviewers. The estimate presented in the table is the mean product, with the minimum and maximum being the product of the lowest within-herd prevalence and lowest herd-level prevalence and the maximum being the product of the highest within-herd prevalence and highest herd-level prevalence.

11 Pooled continent-level prevalence estimates from Loiklung et al. (2022) were converted to incidence using an incidence-prevalence ratio of 2.2 (Duffield et al., 1998; Oetzel, 2004, 2013).

**Table 3:** Estimated pairwise interdisease odds ratios (OR) used as input values in the comorbidity adjustment of dairy cattle disease impacts^1^

OR	Distribution	Mean (parameters)	Source(s)
CK:CM	PERT	2.13 (min = 1.20, max = 3.40)	Dohoo and Martin (1984), Gröhn et al. (1989)^2^
CK:LAM	PERT	1.65 (min = 1.20, max = 2.40)	Dohoo and Martin (1984), Gröhn et al. (1989)^2^
CK:MF	Normal	1.60 (SD = 0.13)	Gröhn et al. (1989)^3^
CK:OC	PERT	1.97 (min = 1.30, max = 4.10)	Dohoo and Martin (1984), Gröhn et al. (1989), Gröhn et al. (1990)^3^
CK:RP	PERT	1.55 (min = 1.00, max = 1.90)	Dohoo and Martin (1984), Gröhn et al. (1989)^2^
CK:SCK	Normal	6.95 (SD = 1.28)	Raboisson et al. (2014)^3^
CK:SCM	Normal	2.40 (SD = 0.41)	Gröhn et al. (1989)^3^
CM:LAM	Fixed	2.10	Dohoo and Martin (1984)
CM:PTB	Normal	1.89 (SD = 0.20)	Rossi et al. (2017)^3^
CM:RP	Normal	2.70 (SD = 0.33)	Suthar et al. (2013)^3^
CM:SCK	Normal	1.64 (SD = 0.20)	Raboisson et al. (2014)
CM:SCM	PERT	3.05 (min = 1.30, max = 6.50)	van den Borne et al. (2011)^2^
DA:CM	PERT	3.45 (min = 1.40, max = 4.80)	Dohoo and Martin (1984), Gröhn et al. (1989)^2^
DA:MF	Normal	2.50 (SD = 0.48)	Gröhn et al. (1989)^3^
DA:RP	PERT	3.50 (min = 1.60, max = 4.60)	Dohoo and Martin (1984), Gröhn et al. (1989)^2^
DA:SCK	Normal	3.87 (SD = 0.34)	Raboisson et al. (2014)^3^
DA:SCM	Normal	3.60 (SD = 1.35)	Gröhn et al. (1989)^3^
DYS:LAM	Normal	2.09 (SD = 0.26)	Malašauskienė et al. (2022)^3^
DYS:OC	Fixed	0.40	Dohoo and Martin (1984)
DYS:RP	PERT	2.74 (min = 1.25, max = 5.96)	Erb et al. (1981a), Gröhn et al. (1990), Kumari et al. (2015), Roche et al. (2023)^2^
LAM:OC	Normal	2.63 (SD = 1.44)	Melendez et al. (2003)^3^
LAM:PTB	Normal	2.70 (SD = 1.22)	Smith and van Winden (2019)^3^
LAM:RP	Normal	1.50 (SD = 0.31)	Suthar et al. (2013)^3^
LAM:SCK	Normal	2.01 (SD = 0.20)	Raboisson et al. (2014)^3^
MET:CK	PERT	2.42 (min = 1.20, max = 10.4)	Gröhn et al. (1989), Kaneene and Miller (1995), Suthar et al. (2013)^2^
MET:CM	PERT	2.30 (min = 1.20, max = 3.8)	Gröhn et al. (1989), Suthar et al. (2013)^2^
MET:DA	PERT	3.40 (min = 1.60, max = 7.60)	Gröhn et al. (1989), Gröhn et al. (1990)^2^
MET:DYS	PERT	2.95 (min = 0.98, max = 9.72)	Erb et al. (1981a), Gröhn et al. (1990), Kaneene and Miller (1995), Giuliodori et al. (2013), Mahnani et al. (2015), Kumari et al. (2016)^2^
MET:LAM	Normal	6.10 (SD = 1.45)	Gröhn et al. (1990)^3^
MET:MF	Normal	1.50 (SD = 0.15)	Gröhn et al. (1990)^3^
MET:OC	PERT	1.94 (min = 1.20, max = 3.00)	Gröhn et al. (1989), Gröhn et al. (1990)^2^
MET:RP	PERT	3.53 (min = 1.8, max = 6.52)	Gröhn et al. (1990), Kaneene and Miller (1995), Suthar et al. (2013)^2^
MET:SCK	Normal	1.94 (SD = 0.09)	Raboisson et al. (2014)^3^
MF:DYS	Normal	9.70 (SD = 1.30)	Gröhn et al. (1990)^3^
MF:LAM	Fixed	3.60	Dohoo and Martin (1984)
MF:RP	Normal	2.40 (SD = 0.20)	Gröhn et al. (1990)^3^
RP:OC	PERT	2.18 (min = 1.78, max = 2.57)	Erb et al. (1981a)^2^
SCK:RP	Normal	1.52 (SD = 0.19)	Raboisson et al. (2014)^3^

1 Mean estimates are followed by distribution parameters in parentheses, unless the value was assumed to be fixed. Detailed information, including underlying assumptions, is available in Supplemental File S2 (see Notes). Odds ratios are listed alphabetically. CK = clinical ketosis; CM = clinical mastitis; DA = displaced abomasum; DYS = dystocia; LAM = lameness; MET = metritis; MF = milk fever; PTB = paratuberculosis; RP = retained placenta; OC = ovarian cyst; SCK = subclinical ketosis; SCM = subclinical mastitis.

2 Average of reported values. Minimum (min) and maximum (max) are the range of estimates.

3 Standard deviation approximated from confidence interval.

**Table 4:** Estimated disease-specific impacts on annual milk yield, calving interval, and premature culling risk used as input values in the comorbidity adjustment, as described in Rasmussen et al. (2022b)^1^

Disease	Yield impact (% decrease in annual milk/cow)			Fertility impact (% increase in calving interval)			Culling impact (culling hazard ratio)		
	Distribution	Mean (parameters)	Source(s)	Distribution	Mean (parameters)	Source(s)	Distribution	Mean (parameters)	Source(s)
CK	PERT	0.43 (min = 0.24, max = 1.04)	Bareille et al. (2003)^2^	Normal	1.45 (SD = 0.36)	Fourichon et al. (2000)	Normal	1.50 (SD = 0.30)	Probo et al. (2018)^3^
CM	Normal	3.25 (SD = 0.76)	Boujenane et al. (2015), Heikkilä et al. (2018), Fukushima et al. (2022)^3^	Normal	8.42 (SD = 2.42)	Schrick et al. (2001), Klaas et al. (2004), Gunay and Gunay (2008), Ahmadzadeh et al. (2009), Peake et al. (2011), Boujenane et al. (2015), Toni et al. (2015), Mellado et al. (2018), Salar et al. (2019), Campos et al. (2020), Fukushima et al. (2022), Ramos et al. (2022)^3^	Normal	2.30 (SD = 0.31)	Sharifi et al. (2013), Haine et al. (2017), Hertl et al. (2018), Cruz et al. (2021), Fukushima et al. (2022)^3^
DA	PERT	2.84 (min = −1.45, max = 9.19)	Jorritsma et al. (2008), Fiore et al. (2018)^2^	Normal	1.08 (SD = 2.04)	Fourichon et al. (2000), Jorritsma et al. (2008), Toni et al. (2015)^3^	PERT	2.85 (min = 1.00, max = 7.90)	Jorritsma et al. (2008), Sharifi et al. (2013), Probo et al. (2018)^4^
DYS	Normal	4.92 (SD = 0.97)	Barrier and Haskell (2011), Gaafar et al. (2011), Atashi et al. (2012a), Atashi et al. (2012b), Hossein-Zadeh (2014), Fernandes et al. (2021), Malašauskienė et al. (2022), Roche et al. (2023)^3^	Normal	2.40 (SD = 0.93)	Fourichon et al. (2000), Gaafar et al. (2011), Hayes et al. (2012), Hossein-Zadeh (2014)^3^	PERT	1.26 (min = 0.60, max = 2.10)	López de Maturana et al. (2007), Hayes et al. (2012), Ghavi Hossein-Zadeh (2016), Roche et al. (2023)^5^
LAM	Normal	4.81 (SD = 0.87)	Green et al. (2002), Hernandez et al. (2002), Kocak and Ekiz (2006), Amory et al. (2008), King et al. (2017), Puerto et al. (2021), Malašauskienė et al. (2022), Prasomsri (2022)^3^	PERT	3.30 (min = 1.19, max = 10.71)	Fourichon et al. (2000), Peake et al. (2011), Toni et al. (2015), Mellado et al. (2018)^2^	Normal	1.74 (SD = 0.17)	Booth et al. (2004), Sharifi et al. (2013), Cruz et al. (2021)^3^
MET	Normal	5.61 (SD = 1.35)	Giuliodori et al. (2013), Dawod and Min (2014), Mahnani et al. (2015), Kumari et al. (2016), Piccardi et al. (2016)^3^	Normal	14.67 (SD = 8.54)	Fourichon et al. (2000), Dawod and Min (2014), Mahnani et al. (2015), Toni et al. (2015), Kumari et al. (2016), Mellado et al. (2018)^3^	Normal	1.05 (SD = 0.15)	Probo et al. (2018)^3^
MF	Fixed	0.54	Bareille et al. (2003)	PERT	2.41 (min = 2.03, max = 3.10)	Fourichon et al. (2000), Hayes et al. (2012)^2^	Normal	3.00 (SD = 0.90)	Hayes et al. (2012), Probo et al. (2018)^3^
OC	PERT	3.75 (min = 1.71, max = 4.33)	Erb et al. (1981a, 1981b), Erb et al. (1985), Bigras-Poulin et al. (1990)^6^	PERT	9.69 (min = 5.04, max = 21.43)	Fourichon et al. (2000), Klaas et al. (2004), Toni et al. (2015)^2^	Normal	1.62 (SD = 0.42)	Sharifi et al. (2013)
PTB	Normal	4.30 (SD = 0.67)	McAloon et al. (2016)^7^	Normal	5.35 (SD = 2.53)	Johnson-Ifearulundu et al. (2000), Jurkovich et al. (2016), Ózsvari et al. (2020)^3^	Normal	2.31 (SD = 0.35)	Hendrick et al. (2005), Tiwari et al. (2005), Raizman et al. (2009)^3^
RP	Normal	4.20 (SD = 1 0.15)	Kumari et al. (2015), Mahnani et al. (2021)^3^	Normal	6.76 (SD = 1.56)	Fourichon et al. (2000), Könyves et al. (2009), Hayes et al. (2012), Kumari et al. (2015), Toni et al. (2015), Mahnani et al. (2021), Kamel et al. (2022), Ramos et al. (2022)^3^	Normal	1.60 (SD = 0.32)	Hayes et al. (2012), Probo et al. (2018)^3^
SCK	Normal	8.40 (SD = 1.19)	Raboisson et al. (2014)	Normal	1.12 (SD = 1.82)	Fourichon et al. (2000), Toni et al. (2015)^3^	Normal	1.92 (SD = 0.18)	Raboisson et al. (2014)
SCM	Normal	6.29 (SD = 1.20)	Pfützner and Ózsvari (2017), Bagri et al. (2018), Heikkilä et al. (2018), Martins et al. (2020), Fernandes et al. (2021)^3^	PERT	0.26 (min = −0.12, max = 5.68)	Fourichon et al. (2000), Schrick et al. (2001), Klaas et al. (2004), Peake et al. (2011)^2^	Normal	1.45 (SD = 0.25)	Beaudeau et al. (1995)^3^

1 Detailed information (including underlying assumptions) is available in Supplemental File S3 (see Notes), and meta-analysis results (including forest plots) are available in Supplemental File S4 (see Notes). Diseases are listed in alphabetical order. CK = clinical ketosis; CM = clinical mastitis; DA = displaced abomasum; DYS = dystocia; LAM = lameness; MET = metritis; MF = milk fever; PTB = paratuberculosis; RP = retained placenta; OC = ovarian cyst; SCK = subclinical ketosis; SCM = subclinical mastitis; min = minimum; max = maximum.

2 Sample-weighted average (cases). Minimum and maximum are the range of estimates.

3 Meta-analysis of means, inverse variance method, and heterogeneity evaluated using Paule-Mandel weighting (Paule and Mandel, 1982). Random-effects estimate.

4 Sample-weighted average (study). Minimum and maximum are the range of estimates.

5 Average across studies. Minimum and maximum are the range of estimates.

6 Adapted from Fourichon et al. (1999). Sample-weighted average (cases). Minimum and maximum are the range of estimates.

7 Initially, it was attempted to combine the results from the McAloon et al. (2016) meta-analysis with newer estimates from Jurkovich et al. (2016) and Ózsvari et al. (2020) into a new meta-analysis of means with heterogeneity evaluated using Paule-Mandel weighting (Paule and Mandel, 1982). However, the random-effects estimate was unable to yield stable results due to the ratio of largest to smallest sampling variance being extremely large.

### Comorbidity Adjustment and Economic Analysis

To avoid double counting disease impacts, which would result in overestimating the burden of the diseases being modeled, a framework for comorbidity adjustment was required. Rasmussen et al. (2022b) described a modeling approach using Bayes' theorem, disease probability estimates, disease impact estimates, and interdisease odds ratios (**OR**) to estimate the probability of various disease combinations in a population and adjust impact estimates to reflect comorbidities. Within the approach, disease impacts in the literature are treated as conflations of the impacts of a nest of concurrent diseases and conditions, with the impact estimate being a weighted sum of the products of disease probabilities and disease impacts. Once disease combination probabilities are estimated, the difference between the probability of disease occurrence given the presence of an associated disease and disease occurrence given the absence of an associated disease is used to scale disease impact estimates based on the magnitude of the statistical associations (i.e., OR) relating disease pairs. Although in the model's illustration in Rasmussen et al. (2022b) it was assumed that prevalence and incidence were roughly equivalent, this assumption failed to capture that the probability of occurrence for diseases with relatively short durations (i.e., durations less than a lactation) will be underestimated by prevalence estimates. Therefore, in the current study, lactational incidence rates were converted to lactational disease probabilities assuming that discrete disease events are Poisson-distributed, such that

**Figure 2:**
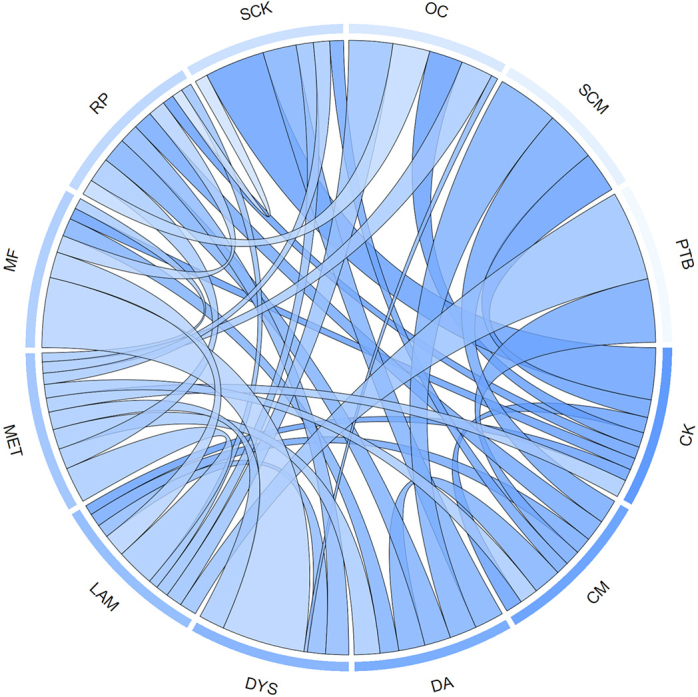
Graphical representation of disease associations used in the comorbidity adjustment of disease impact estimates. Wider connections represent stronger statistical associations between disease pairs. CK = clinical ketosis; CM = clinical mastitis; DA = displaced abomasum; DYS = dystocia; LAM = lameness; MET = metritis; MF = milk fever; PTB = paratuberculosis; RP = retained placenta; OC = ovarian cyst; SCK = subclinical ketosis; SCM = subclinical mastitis.

(1) Pr(*x*) = 1 – *e*^−I^ ,

where Pr(*x*) is the probability of a case of disease *x* within a lactation and I is the lactational incidence rate of the disease. For lifelong chronic diseases, such as PTB, prevalence was used instead of incidence, as prevalence accurately reflects the probability of infected animals within the herd within a lactation. The OR capturing statistical associations between the modeled diseases are summarized graphically in Figure 2 and described in detail in Table 3.

Monte Carlo methods, which involve repeated random sampling to estimate a range of possible outcomes, were used to estimate the comorbidity-adjust impacts of the diseases modeled and their resulting economic losses. A single 50,000-iteration Monte Carlo analysis was first run to estimate the comorbidity-adjusted disease impacts based on the prevalence or incidence estimates, OR, and pooled disease impact estimates described in Tables 2, 3, and 4, respectively. Because the OR and impact estimates were collected from studies across a wide array of countries, it was assumed that these impacts and interdisease associations were representative of the global dairy cattle population, and therefore, the global average prevalence or incidence for each disease, weighted by national herd sizes, was used as a baseline for comorbidity adjustment using the method described in Rasmussen et al. (2022b). Once the comorbidity-adjusted disease impacts were estimated (Table 5), a set of 50,000-iteration Monte Carlo simulations were run, with a single 50,000-iteration simulation for each of the 183 countries modeled, randomly sampling from country-specific prevalence or incidence estimates and the distributions estimated for disease impacts. These 183 Monte Carlo simulations generated country-specific loss estimates. Monte Carlo methods have been used in analyses of mastitis (Green et al., 2004; Steeneveld et al., 2011), neosporosis (Häsler et al., 2006), classical swine fever (Karsten et al., 2005), PTB (Kudahl et al., 2007; Rasmussen et al., 2021b,c, 2022a) and foot-and-mouth disease (Horst et al., 1999; Bates et al., 2003; Branscum et al., 2007; Beyi, 2012; Rasmussen et al., 2024). Lastly, the sensitivity of estimated losses per cow in an average country to variations in input values was assessed by varying the mean input values by ± 20% of their mean with all else constant.

**Table 5:** Mean estimated comorbidity-adjusted disease impacts used in the estimation of global economic losses and the percent change in their estimated mean values (Table 4) due to comorbidity adjustment^1^

Disease^2^	Yield impact (% decrease in annual milk/cow)		Fertility impact (% increase in calving interval)		Culling impact (culling hazard ratio)	
	Mean adjusted impact	Change from unadjusted (%)	Mean adjusted impact	Change from unadjusted (%)	Mean adjusted impact	Change from unadjusted (%)
CK	0.03	−93.02	0.34	−76.55	1.18	−21.33
CM	1.36	−58.15	6.09	−27.67	1.90	−17.39
DA	1.18	−58.45	0.78	−27.78	2.75	−3.51
DYS	3.48	−29.27	1.11	−53.75	1.18	−6.35
LAM	2.62	−45.53	1.86	−43.64	1.40	−19.54
MET	2.87	−48.84	11.22	−23.52	1.03	−1.90
MF	0.07	−87.04	1.06	−56.02	2.64	−12.00
OC	2.59	−30.93	9.03	−6.81	1.51	−6.79
PTB	3.37	−21.63	4.23	−20.93	2.07	−10.39
RP	2.30	−45.24	3.74	−44.67	1.29	−19.38
SCK	7.11	−15.36	0.39	−65.18	1.67	−13.02
SCM	5.58	−11.29	0.04	−84.62	1.25	−13.79

1 Based on a 50,000-iteration Monte Carlo simulation for each impact type (i.e., yield, fertility, culling) using estimated global average disease prevalence or incidence (Table 2) and estimated interdisease odds ratios (Table 3).

2 Diseases are listed in alphabetical order. CK = clinical ketosis; CM = clinical mastitis; DA = displaced abomasum; DYS = dystocia; LAM = lameness; MET = metritis; MF = milk fever; PTB = paratuberculosis; RP = retained placenta; OC = ovarian cyst; SCK = subclinical ketosis; SCM = subclinical mastitis.

### Statistical Software and Packages

All statistical analyses, modeling, and plotting were implemented in R (version 4.3.1; R Core Team, 2023) using RStudio (RStudio Team, 2023) with the following packages: Cairo (Urbanek and Horner, 2023), circlize (Gu et al., 2014), data.table (Urbanek and Horner, 2023), dplyr (Wickham et al., 2023a), forcats (Wickham, 2023a), geodata (Hijmans et al., 2023), ggplot2 (Wickham, 2016), ggspatial (Dunnington, 2023), Hmisc (Harrell, 2023), magrittr (Bache and Wickham, 2022), mc2d (Pouillot and Delignette-Muller, 2010), meta (Balduzzi et al., 2019), (Ooms, 2023), paletteer (Hvitfeldt, 2021), prevalence (Devleesschauwer et al., 2022), openxlsx (Schauberger, 2023), rjags (Plummer et al., 2023), sf (Pebesma, 2018), stringr (Wickham, 2023b), and tidyr (Wickham et al., 2023b).

## RESULTS

**Figure 3:**
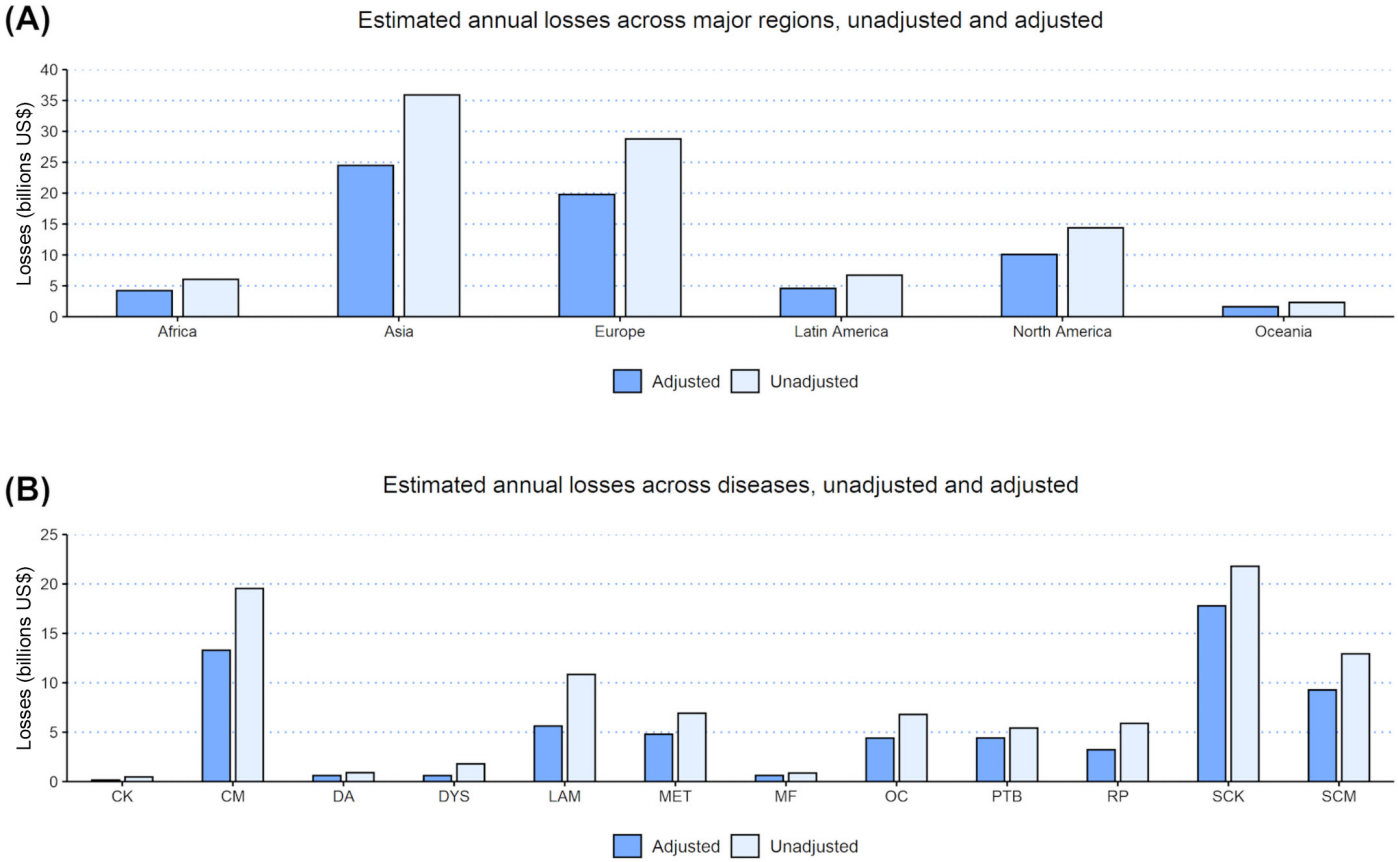
Estimated global losses due to dairy cattle diseases across regions and diseases. (A) Total annual losses across regions. (B) Total annual losses across diseases. See Supplemental File S5 for details. CK = clinical ketosis; CM = clinical mastitis; DA = displaced abomasum; DYS = dystocia; LAM = lameness; MET = metritis; MF = milk fever; PTB = paratuberculosis; RP = retained placenta; OC = ovarian cyst; SCK = subclinical ketosis; SCM = subclinical mastitis.

Without comorbidity adjustment, aggregate annual regional losses ranged from US$2.30B in Oceania to US$35.90B in Asia (Figure 3A and Supplemental File S5, see Notes). With comorbidity adjustment, aggregate losses decreased, ranging from US$1.61B to US$24.50B in Oceania and Asia, respectively. Overall, when statistical associations between diseases were disregarded (i.e., without adjustment for comorbidities), mean total global losses due to all diseases modeled would have been overestimated by 45% (US$64.74B when adjusted compared with US$94.12B without adjustment), equivalent to a 29% reduction in aggregate annual losses due to adjustment. Across diseases, for both unadjusted and adjusted annual global losses, CK and SCK were the least and most costly diseases, with adjusted losses of US$0.15B to US$17.78B, for CK and SCK, respectively (Figure 3B and Supplemental File S5). The most impactful comorbidity adjustments, in terms of their reduction in economic losses due to disease (Figure 3), were to CK, DYS, LAM, and OC, with reductions of approximately 69%, 67%, 48%, and 35%, respectively.

**Figure 4:**
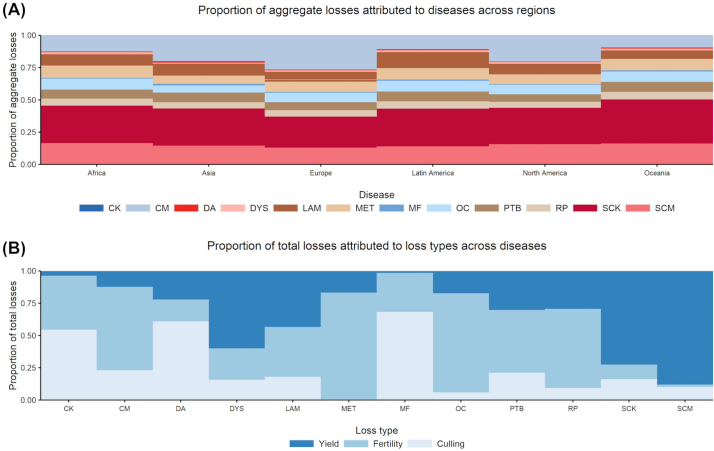
Proportion of total losses attributed to diseases across regions and loss types (reduced milk yield, reduced fertility, and increased culling). (A) Diseases across regions. (B) Loss types across diseases. See Supplemental Files S6 and S7 for details. CK = clinical ketosis; CM = clinical mastitis; DA = displaced abomasum; DYS = dystocia; LAM = lameness; MET = metritis; MF = milk fever; PTB = paratuberculosis; RP = retained placenta; OC = ovarian cyst; SCK = Subclinical ketosis; SCM = Subclinical mastitis.

The proportion of total comorbidity-adjusted losses attributable to the diseases modeled varied across regions (Figure 4A; Supplemental Files S6 and S7, see Notes). For example, although SCK was estimated to be the costliest disease overall, it accounted for as much as 35% of losses in Oceania, where CM accounted for less than 10%, and as little as 24% of losses in Europe, where CM accounted for 27%. However, globally, the greatest proportions of losses were attributable to SCK (a proportion of 0.27 of global losses), CM (0.21), and SCM (0.14), followed by LAM (0.09), MET (0.07), PTB (0.07), and OC (0.07). Similarly, the proportion of total losses attributable to loss types (i.e., reduced yield, reduced fertility, and increased culling) varied across diseases (Figure 4B; Supplemental File S7, see Notes). For example, at the mean level, it was estimated that SCM (a proportion of 0.02 of total losses) and SCK (0.11) resulted in little losses due to reduced fertility, whereas a significant proportion of total losses due to MET (0.83), OC (0.77), RP (0.61), and PTB (0.49) were attributable to reduced fertility.

**Figure 5:**
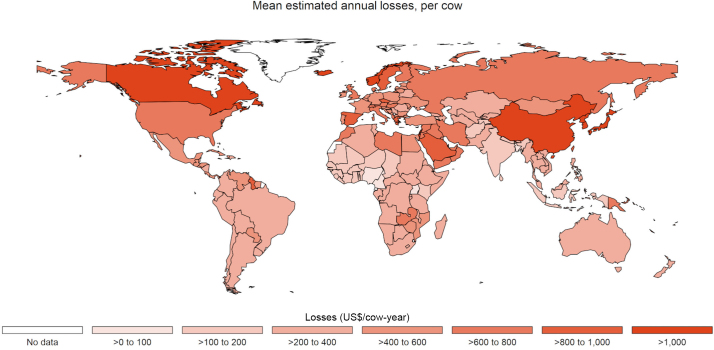
Total comorbidity-adjusted annual losses per cow due to mastitis (subclinical and clinical), lameness, paratuberculosis (Johne's disease), displaced abomasum, dystocia, metritis, milk fever, ovarian cysts, retained placenta, and ketosis (subclinical and clinical). See Supplemental File S8 for details.

**Figure 6:**
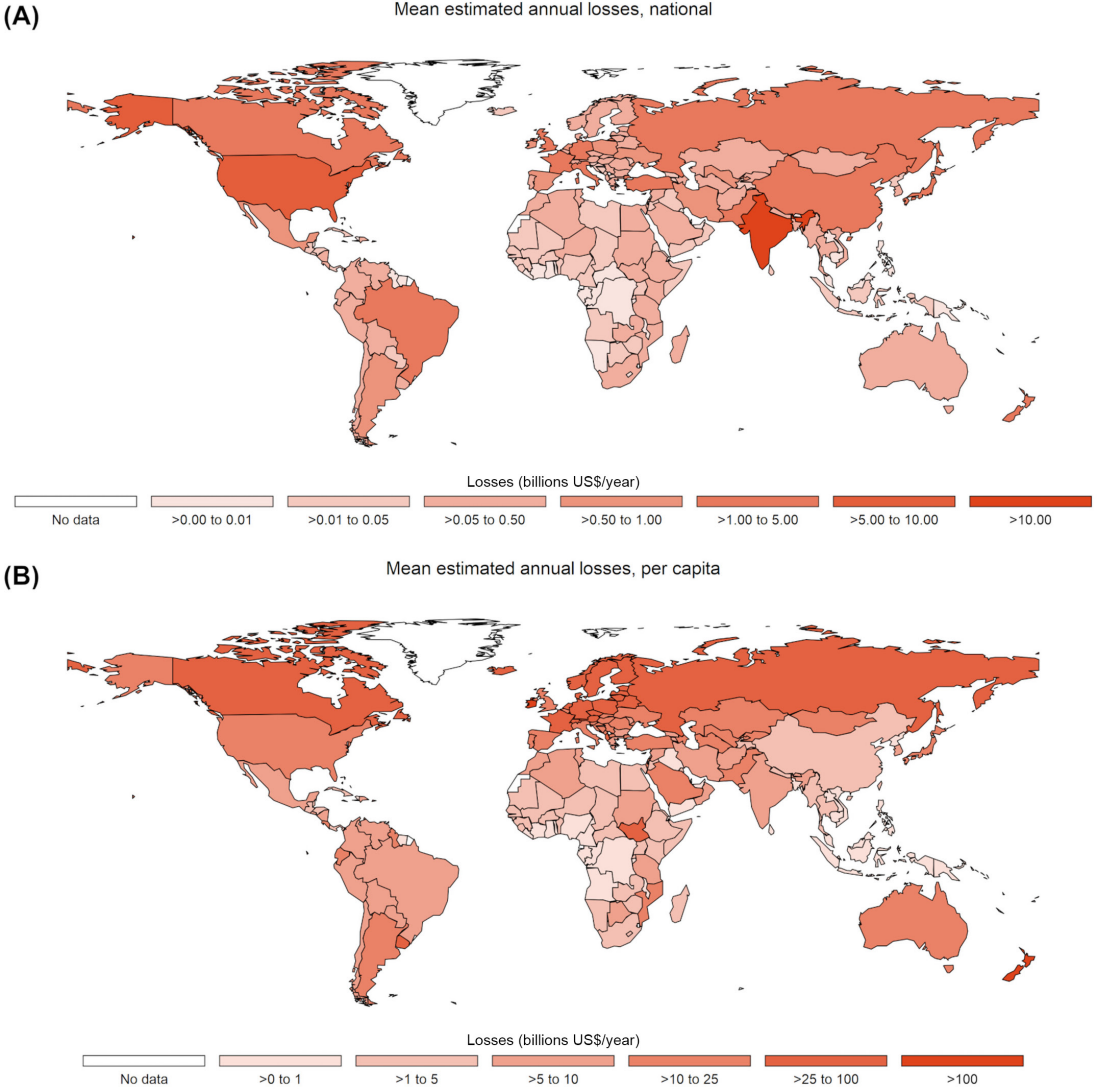
Mean estimated comorbidity-adjusted losses due to mastitis (subclinical and clinical), lameness, paratuberculosis (Johne's disease), displaced abomasum, dystocia, metritis, milk fever, ovarian cysts, retained placenta, and ketosis (subclinical and clinical) among global dairy cattle. (A) Annual national losses. See Supplemental File S9 for details. (B) Annual losses per capita. Population data obtained FAO (FAOSTAT, 2023a). See Supplemental File S10 for details.

Across the 183 countries modeled, comorbidity-adjusted total annual losses per cow (Figure 5 and Supplemental File S8, see Notes) ranged from US$72 in Nigeria to US$1,900 in South Korea (Supplemental File S8, see Notes), with a global cow-weighted average of US$351 (Table 6). When per-cow losses were aggregated using estimated national herds (Figure 6A and Supplemental Files S9 and S10; see Notes), total annual national losses ranged from just US$5,200 in Seychelles to US$12B in India (Supplemental File S9, see Notes). Estimated losses in the United States were comparable to those in India at US$8B, followed by losses of US$5B and US$4B in China and Russia, respectively. However, when human population was considered (Figure 6B), the most affected countries were New Zealand (US$220/person-year), Ireland (US$140/person-year), and Denmark (US$70/person-year), whereas India, the United States, China, and Russia went from the top 4 most affected countries to the 72nd, 27th, and 111th, and 23rd most affected countries, respectively (Supplemental File S10, see Notes). Considering losses as a percentage of gross domestic product (**GDP**) and as a percentage of milk revenue (i.e., the product of national milk production and the price of milk) resulted in similarly varied rankings in terms of relative national impact. Detailed country-level results for losses as a percentage of GDP, losses per capita, and losses as a percentage of gross milk revenue are available in Supplemental Files S10 and S11 (see Notes).

**Table 6:** Estimated regional average comorbidity-adjusted annual losses per cow (US$) across regions and diseases, with regional averages weighted by national herd size (Table 1)^1^

Disease^2^	Africa	Asia	Europe	Latin America	North America	Oceania	Global
CK	0.44 (0.66)	0.69 (0.83)	1.11 (1.06)	0.79 (0.89)	1.18 (1.09)	0.72 (0.85)	0.79 (0.89)
CM	32.25 (5.68)	47.44 (6.89)	151.54 (12.31)	35.77 (5.98)	153.99 (12.41)	27.40 (5.23)	72.13 (8.49)
DA	1.74 (1.32)	3.01 (1.74)	4.41 (2.10)	3.38 (1.84)	4.56 (2.14)	3.05 (1.75)	3.31 (1.82)
DYS	4.24 (2.06)	1.98 (1.41)	3.62 (1.90)	4.55 (2.13)	10.05 (3.17)	3.53 (1.88)	3.29 (1.81)
LAM	23.02 (4.80)	22.02 (4.69)	43.81 (6.62)	41.94 (6.48)	61.64 (7.85)	20.23 (4.50)	30.48 (5.52)
MET	25.77 (5.08)	15.46 (3.93)	42.57 (6.52)	30.41 (5.51)	58.53 (7.65)	26.03 (5.10)	25.98 (5.10)
MF	1.63 (1.28)	3.12 (1.77)	4.39 (2.10)	3.36 (1.83)	4.33 (2.08)	2.99 (1.73)	3.34 (1.83)
OC	23.58 (4.86)	13.65 (3.70)	40.4 (6.36)	27.4 (5.23)	55.76 (7.47)	23.76 (4.87)	23.9 (4.89)
PTB	17.86 (4.23)	17.93 (4.23)	35.92 (5.99)	25.84 (5.08)	44.16 (6.65)	23.39 (4.84)	23.96 (4.89)
RP	15.04 (3.88)	11.7 (3.42)	27.47 (5.24)	19.44 (4.41)	35.89 (5.99)	17.49 (4.18)	17.45 (4.18)
SCK	77.23 (8.79)	69.5 (8.34)	136.45 (11.68)	99.56 (9.98)	216.17 (14.7)	102.28 (10.11)	96.52 (9.82)
SCM	44.36 (6.66)	35.07 (5.92)	72.54 (8.52)	47.60 (6.90)	119.38 (10.93)	48.57 (6.97)	50.33 (7.09)
Total	267.16 (26.58)	241.58 (17.19)	564.23 (49.74)	340.04 (18.88)	765.64 (38.00)	299.44 (14.81)	351.47 (28.80)

1 Mean regional values followed by SD in parentheses. Unadjusted estimates are available in Supplemental File S8.

2 Diseases are listed in alphabetical order. CK = clinical ketosis; CM = clinical mastitis; DA = displaced abomasum; DYS = dystocia; LAM = lameness; MET = metritis; MF = milk fever; PTB = paratuberculosis; RP = retained placenta; OC = ovarian cyst; SCK = subclinical ketosis; SCM = subclinical mastitis.

**Figure 7:**
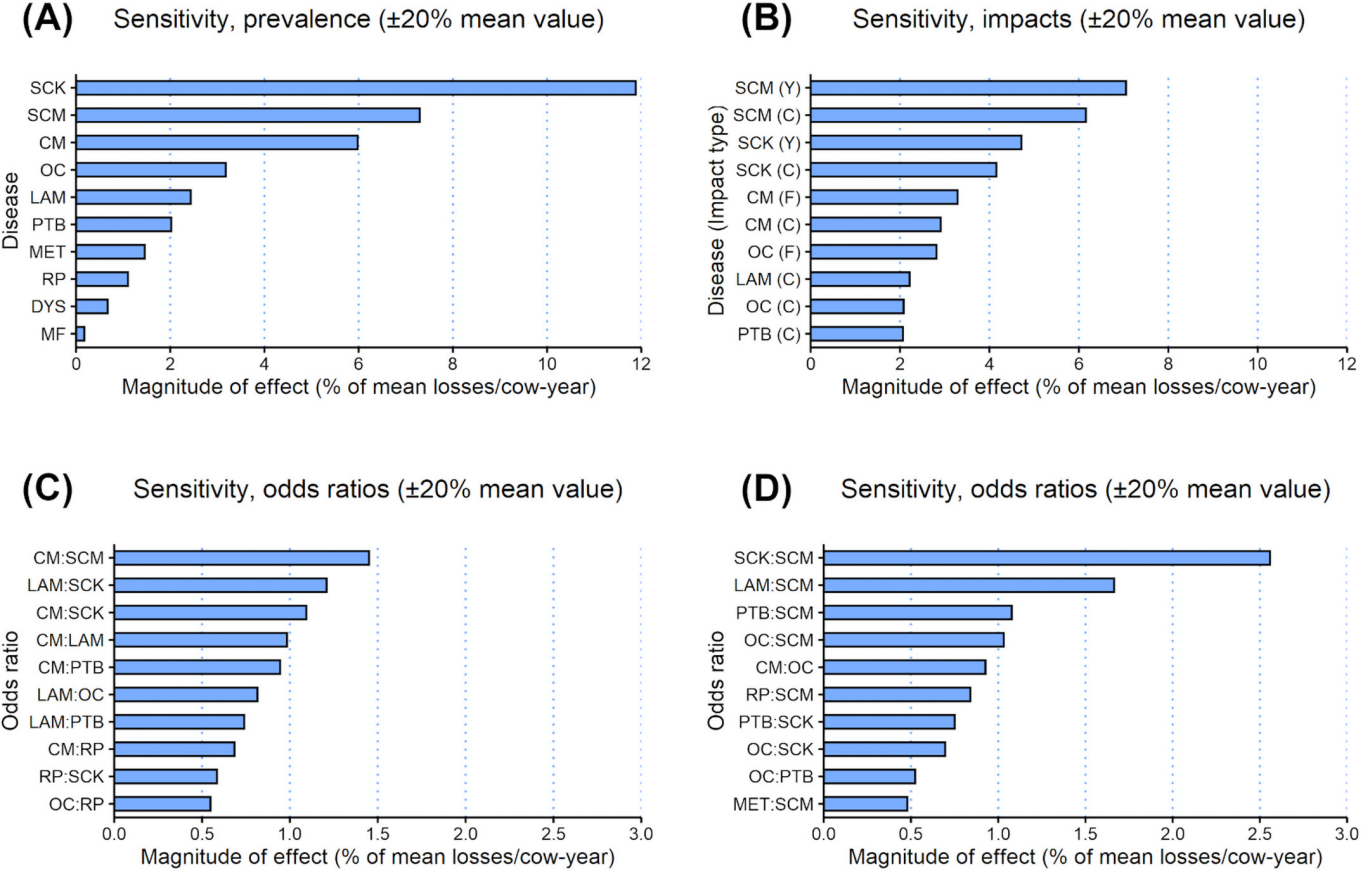
Sensitivity of total estimated annual losses per cow in an average country to variations in the model's input values. (A) Variations in disease prevalence (paratuberculosis) and incidence (all other diseases modeled). (B) Variations in disease impacts. Y = yield impact; F = fertility impact; C = culling impact. (C) Variations in the magnitude of interdisease odds ratios (OR) that were captured in the literature search (i.e., mean OR ≠ 1). (D) Variations in the magnitude of interdisease odds ratios that were not captured in the literature search (i.e., mean OR = 1). CK = clinical ketosis; CM = clinical mastitis; DA = displaced abomasum; DYS = dystocia; LAM = lameness; MET = metritis; MF = milk fever; PTB = paratuberculosis; RP = retained placenta; OC = ovarian cyst; SCK = subclinical ketosis; SCM = subclinical mastitis.

Sensitivity analyses revealed that, among prevalence or incidence values (Figure 7A), variations in the incidence of SCK, SCM, and CM were the most impactful in terms of their effects on estimated losses. Similarly, among disease impacts on yield, fertility, and culling, estimated losses were most sensitive to variations in the yield and culling impacts of SCM and SCK, and the fertility and culling impacts of CM (Figure 7B). Among interdisease OR whose means were assumed to be ≠ 1 (i.e., OR for which estimates were found in the literature), estimated losses were most sensitive to variations in the strength of association between CM and SCM, LAM and SCK, and CM and SCK, with variations in the OR relating CM and LAM and CM and PTB being comparably impactful to the latter (Figure 7C). Among interdisease OR for which no estimates were found in the literature, associations between SCK and SCM, LAM and SCM, and PTB and SCM were identified as being the most potentially impactful (Figure 7D).

## DISCUSSION

The current study combined prevalence or incidence estimates, data on herd characteristics, estimates of statistical associations between diseases, and disease impact estimates to assess the global losses due to 12 dairy cattle diseases, adjusted for comorbidity, across 183 milk-producing countries. It was estimated that these diseases result in annual losses per cow (Table 6) and annual national losses (Table 7 and Supplemental File S5) of approximately US$351/cow-year and US$65B/year, respectively. Comorbidity adjustment mitigated a 45% overestimation of aggregate losses, which were estimated to be US$94B when associations between diseases were ignored. Although the greatest aggregate annual losses were estimated to be in India, the United States, and China, all countries with annual losses close to or exceeding US$5B per year, depending on the measure of losses used (losses as a percent of GDP, losses per capita, losses as a percent of milk revenue), the relative economic burden of these dairy cattle diseases varied markedly. Comorbidity-adjusted losses were equivalent to average global losses of approximately US$12 per person-year in milk-producing countries, with the greatest mean per capita losses being in New Zealand (US$220/person-year), Ireland (US$140/person-year), and Denmark (US$70/person-year).

**Table 7:** Estimated comorbidity-adjusted annual losses (US$, in billions) across regions and diseases^1^

Disease^2^	Africa	Asia	Europe	Latin America	North America	Oceania	Global
CK	0.01 (0.01)	0.07 (0.08)	0.04 (0.04)	0.01 (0.01)	0.02 (0.01)	<0.01 (<0.01)	0.15 (0.16)
CM	0.51 (0.09)	4.81 (0.70)	5.31 (0.43)	0.48 (0.08)	2.02 (0.16)	0.15 (0.03)	13.29 (1.56)
DA	0.03 (0.02)	0.31 (0.18)	0.15 (0.07)	0.05 (0.02)	0.06 (0.03)	0.02 (0.01)	0.61 (0.34)
DYS	0.07 (0.03)	0.20 (0.14)	0.13 (0.07)	0.06 (0.03)	0.13 (0.04)	0.02 (0.01)	0.61 (0.33)
LAM	0.36 (0.08)	2.23 (0.48)	1.54 (0.23)	0.56 (0.09)	0.81 (0.10)	0.11 (0.02)	5.62 (1.02)
MET	0.41 (0.08)	1.57 (0.40)	1.49 (0.23)	0.41 (0.07)	0.77 (0.10)	0.14 (0.03)	4.79 (0.94)
MF	0.03 (0.02)	0.32 (0.18)	0.15 (0.07)	0.05 (0.02)	0.06 (0.03)	0.02 (0.01)	0.61 (0.34)
OC	0.37 (0.08)	1.38 (0.37)	1.42 (0.22)	0.37 (0.07)	0.73 (0.10)	0.13 (0.03)	4.40 (0.90)
PTB	0.28 (0.07)	1.82 (0.43)	1.26 (0.21)	0.35 (0.07)	0.58 (0.09)	0.13 (0.03)	4.41 (0.90)
RP	0.24 (0.06)	1.19 (0.35)	0.96 (0.18)	0.26 (0.06)	0.47 (0.08)	0.09 (0.02)	3.21 (0.77)
SCK	1.22 (0.14)	7.05 (0.85)	4.78 (0.41)	1.34 (0.13)	2.84 (0.19)	0.55 (0.05)	17.78 (1.81)
SCM	0.70 (0.10)	3.56 (0.60)	2.54 (0.30)	0.64 (0.09)	1.57 (0.14)	0.26 (0.04)	9.27 (1.31)
Total	4.21 (0.42)	24.50 (1.74)	19.79 (1.74)	4.57 (0.25)	10.07 (0.5)	1.61 (0.08)	64.74 (5.31)

1 Mean values followed by SD in parentheses. Unadjusted estimates are available in Supplemental File S5.

2 Diseases are listed in alphabetical order. CK = clinical ketosis; CM = clinical mastitis; DA = displaced abomasum; DYS = dystocia; LAM = lameness; MET = metritis; MF = milk fever; PTB = paratuberculosis; RP = retained placenta; OC = ovarian cyst; SCK = subclinical ketosis; SCM = subclinical mastitis.

To the authors' knowledge, few global economic analyses of dairy cattle diseases exist. Although exploring databases (e.g., Google Scholar, PubMed, and Web of Science) using combinations and variations of some or all of the keywords “global,” “dairy,” “cattle,” “economic,” and “losses” yielded several global prevalence or incidence literature reviews and meta-analyses, many of which were used to populate the models in this current study, the exploration identified only a single explicitly global economic analysis of losses due to a cattle disease (Reichel et al., 2013). Reichel et al. (2013) combined 25 papers from the beef industry and 72 papers from the dairy industry to estimate the global economic impact of *Neospora caninum*, a coccidian parasite passed in the feces of canids that can cause abortions in cattle (Dubey et al., 2007). This suggests that economic analyses of dairy cattle diseases at the global scale are, at the very best, uncommon.

Similarly, multidisease economic analyses of dairy cattle diseases are also uncommon, with only a handful being identified. For example, Bellows et al. (2002) estimated the total yearly cost of female infertility, abortions and stillbirths, DYS, RP, and MET/pyometra in US cattle to range from US$441 million to US$502 million for beef producers and US$473 million to US$484 million for dairy producers, equivalent to aggregate national losses of approximately US$1B annually. Bennett et al. (1999) estimated the value of output losses due to bovine viral diarrhea (**BVD**), fasciolosis, LAM, leptospirosis, and mastitis in the mainland United Kingdom to be between £108 million and £367 million in 1996 prices (their estimate for output losses due to LAM will be discussed in detail later in this section). Rasmussen et al. (2022b) introduced the comorbidity adjustment framework used in this current study. At the same time, that framework was illustrated using a dairy sector loosely based on the United Kingdom, including an economic analysis of 13 diseases and conditions. It was estimated that the diseases and conditions modeled resulted in total comorbidity-adjusted annual per-cow losses of £404 and found that losses would have been 14% to 61% greater without comorbidity adjustment.

Although it is only an illustration and not a thorough economic analysis, Rasmussen et al. (2022b) is one of few multidisease analyses that explicitly addresses the potential for overestimation when economic loss estimates are not adjusted for comorbidities. Raboisson et al. (2014) is another example of comorbidity adjustment, in which the authors aimed to provide an overview of the relationship between SCK and a range of diseases and conditions. The results of that study were later expanded upon in Raboisson et al. (2015), which used stochastic modeling to estimate the mean total cost of SCK adjusted for the disease's associations with left and right abomasal displacements, CK, MET, RP, subclinical endometritis, purulent viral discharge, CM, and LAM.

In Raboisson et al. (2015), it was estimated that the mean total costs per case of SCK were €257 per calving cow, which can be crudely compared with the average global per-cow losses due to SCK estimated herein (Table 6) using the global average lactational incidence assumed in this current study (Table 2), consumer price index values to adjust for inflation (World Bank, 2023a), and the 2021 €/US$ exchange rate (World Bank, 2023b). This conversion results in comparable mean estimated comorbidity-adjusted per-case losses due to SCK of US$294 and US$202 across the Raboisson et al. (2015) study and the current study, respectively. However, although Raboisson et al. (2015) reported that adjusting for the impacts of associated diseases and health conditions mitigated an overestimation of up to 68% in costs, the impact of comorbidity adjustment for SCK was far smaller in this current study. Specifically, only an 18% reduction in losses due to SCK because of adjustment was observed herein, equivalent to a potential overestimation of only 23%. As will be discussed in the coming paragraphs, this type of disagreement between estimates across studies is not unique, with the overall alignment varying widely.

Bennett et al. (1999) estimated the output losses due to several diseases in the mainland United Kingdom, including LAM, for which they estimated losses ranging from £30.1 million to £65.2 million in 1996 prices. Once adjusted for inflation and converted to US$, these losses are equivalent to losses ranging from US$42 million to US$91 million. In the current study, it was estimated that mean adjusted national losses per year due to LAM among greater UK dairy cattle were US$96 million. Ózsvári et al., 2007, Ózsvári et al., 2020 collected the results of several economic analyses of LAM in dairy cattle, decomposing losses in the Netherlands (Dijkhuizen and Morris, 1997) and losses in Hungary (Ózsvari et al., 2007) into specific components of losses. From Dijkhuizen and Morris (1997), losses due to reduced milk, longer calving interval, and premature culling totaled US$20 per cow. Once adjusted for inflation (World Bank, 2023a), these losses are equivalent to US$34 per cow in 2021, less than the adjusted per-cow losses due to LAM of US$41 estimated for Netherlands herein, even though the latter losses have been adjusted for comorbidities. From Ózsvari et al. (2007), per-cow losses due to the same components were estimated to be US$57. Once adjusted for US inflation (World Bank, 2023a) these losses were equivalent to US$89 per cow in 2021, more than the mean comorbidity-adjusted per-cow losses due to LAM estimated for Hungary herein of US$62, but comparable if unadjusted (US$120).

Rasmussen et al. (2021c) estimated losses due to PTB across a selection of major dairy-producing countries. For simplicity, estimates for the US will be used to compare the study's results to those herein. In Rasmussen et al. (2021c), it was estimated that annual losses in US dairy herds due to PTB were approximately US$42 per cow within positive herds, with approximately 50% of herds being MAP-positive. This is roughly equivalent to losses of US$21 per cow, across both infected and noninfected herds, which is less than half the average adjusted losses of US$54 per cow due to PTB estimated for the United States herein. However, although Rasmussen et al. (2021c) considered losses due to reduced salvage value, salvage losses accounted for only 12% of losses in the United States, and the 2021 study did not consider fertility losses due to PTB, which accounted for nearly 50% of the losses estimated in the current study. In addition, despite adjustments for statistical associations with LAM and mastitis reducing the estimated milk yield and fertility impacts of PTB by approximately 20% in the current study, cow-level prevalence in the current study was assumed to be nearly twice as high as assumed in Rasmussen et al. (2021c).

A variety of potential explanations exist for the observed differences in estimated losses across studies, such as methodological differences, changes in herd structures and economic circumstances over time, and varying degrees of generalizability across study results used as input values. Sensitivity analyses revealed that estimated losses were particularly sensitive to variations in the assumed incidence of SCM, SCK, and CM, which were estimated to be the 3 costliest diseases, even after comorbidity adjustment. Therefore, it is highly likely that observed disagreements between the per-cow loss estimates generated in the current study and those generated elsewhere stem from, primarily, differences in disease incidence or prevalence across study populations. Sensitivity analyses also identified several potentially impactful disease associations that may warrant further investigation (Figure 7D). Specifically, the analyses suggested that an association between SCK and SCM could potentially be more impactful on estimated losses than any of the OR currently included in the comorbidity adjustment model, and that associations between LAM and SCM and PTB and SCM, if significant, could markedly reduce estimated losses due to those diseases if aggregated.

It is also important to recognize the weaknesses of this study. This analysis only captured the value of production losses due to the diseases modeled, but not how herd structures and management practices would adapt to the potentially increased yield per cow, decreased calving intervals, and reduced probability of premature culling if these diseases were absent. Because this analysis did not use a dynamic herd model, it fails to capture how, for example, herd age structures would likely change if these diseases and health conditions were eliminated, and that, continuing with this example, some of these losses would likely be offset by the benefits of having a greater proportion of younger animals in the herd (Rasmussen et al., 2021c). This study also failed to capture disease treatment costs, which in some cases may approach or even exceed the productivity losses associated with the disease. For example, losses due to CK were estimated to be negligible relative to the losses due to the other 11 diseases modeled. However, treatment costs for a case of CK have been estimated to be as high as €275/case (Steeneveld et al., 2020), suggesting that treatment costs due to factors such as medication, labor, and diagnostic tests significantly contribute to losses.

Additionally, this economic analysis relied heavily upon disease impact estimates from geographically, climatically, genetically, economically, and temporally diverse study populations. In other words, it was assumed that impact estimates were globally generalizable and could be standardized based on global averages without explicit consideration for variations in impacts across breeds, management practices, technical and allocative efficiencies, scales of production, access to resources, and market circumstances. For example, it is questionable to generalize estimates of premature culling impacts generated from studies in countries that, at the time of the study, had rigid production quotas, as it is conceivable that these production quotas, if sufficiently rigid, would markedly affect producer decisions about culling. Similarly, it is difficult to determine how applicable the estimated losses due to, for example, forgone milk, would be in countries such as Canada that still maintain production quotas in their dairy sectors. However, as discussed in Rasmussen et al. (2021a), Rasmussen et al. (2021b), and Rasmussen et al. (2021c), Canadian milk production generally increases year-over-year while the number of dairy farms decreases, and Canadian producers trade quotas among themselves, suggesting that Canadian producers operate in conditions somewhere between those of a rigid, quota-bound market and a purely competitive market.

This analysis also assumes that, in the absence of these diseases, milk prices would remain unchanged despite improved productivity among global dairy cows. However, this is likely untrue, particularly when considering countries with large economies or large dairy sectors whose productivity levels directly affect world prices through both consumer demand and producer supply. For example, for a country with a large economy and dairy sector, such as the United States, the elimination of the modeled diseases could potentially increase domestic milk supply, causing lower domestic milk prices, reduced import demand, and lower global milk prices. Therefore, valuing forgone productivity using current prices, as was done herein, likely results in an overestimation of economic losses. Additionally, due to a lack of estimates available in the literature, this study assumed that the prevalence of SCM is roughly equivalent to its incidence. This assumption implies that an average case of SCM has a duration approximately equal to a full lactation, which is unlikely. As a result, the incidence of SCM is almost certainly being underestimated in this study, and, therefore, so too are the estimated losses due to the disease. Lastly, although the IFCN data provided input values for dairy-producing countries that account for over 80% of global dairy production, the production characteristics of the 130 countries making up the remaining 20% were approximated using geoeconomically comparable countries. Therefore, as suggested by Figure 1B, estimated losses for these regionally approximated countries should be interpreted with an added degree of caution.

In future iterations and updates to this global analysis, the systematic search for input values will be expanded to not only include databases other than Scopus, but also to target country-level prevalence and incidence estimates, disease treatment costs, and evidence of potential nonadditive, or even nonlinear, interactions between disease impacts. The addition of other dairy cattle diseases, such as brucellosis, BVD, neosporosis, fasciolosis, and infection with gastrointestinal nematodes, will also be explored. Despite the weaknesses discussed, this study is unique in its scope and its attempt to estimate global losses due to multiple diseases among dairy cattle within a single, consistent methodological framework with explicit consideration for comorbidities. The comorbidity adjustment technique described herein is currently being coded into a standalone R package that, once widely available, will likely be improved upon and refined by the wider scientific community. This study not only highlights the importance of considering statistical associations between diseases when estimating animal health burdens, but also reveals key data gaps regarding global dairy cattle herd characteristics, productivity, disease prevalence and incidence, and disease impacts. By estimating the economic burdens due to these diseases and identifying potentially important disease associations, this study and its results will help guide animal health research and policy at the national and global levels and aid producers in their efforts to make economically sound, evidence-based management decisions.

## CONCLUSIONS

Annual global losses due to the included dairy cattle diseases were US$65B, with SCK, CM, and SCM being the costliest diseases modeled, resulting in estimated annual global losses of US$18B, US$13B, and US$9B, respectively. Without comorbidity adjustment, when statistical associations between diseases were disregarded, mean aggregate global losses would have been overestimated by 45%. Although aggregate annual losses were greatest in the India (US$12B), the United States (US$8B), and China (US$5B), depending on the measure of losses used (losses as a percent of GDP, losses per capita, losses as a percent of gross milk revenue), the relative economic burden of these dairy cattle diseases across countries varied markedly.

## References

[bib1] Abera D. (2017). Management of dystocia cases in the cattle: A review. J. Reprod. Infertil..

[bib2] Adams A.E., Lombard J.E., Fossler C.P., Román-Muñiz I.N., Kopral C.A. (2017). Associations between housing and management practices and the prevalence of lameness, hock lesions, and thin cows on US dairy operations. J. Dairy Sci..

[bib3] Afonso J.S., Bruce M., Keating P., Raboisson D., Clough H., Oikonomou G., Rushton J. (2020). Profiling detection and classification of lameness methods in british dairy cattle research: A systematic review and meta-analysis. Front. Vet. Sci..

[bib4] Ahmadzadeh A., Frago F., Shafii B., Dalton J.C., Price W.J., McGuire M.A. (2009). Effect of clinical mastitis and other diseases on reproductive performance of Holstein cows. Anim. Reprod. Sci..

[bib5] Amory J.R., Barker Z.E., Wright J.L., Mason S.A., Blowey R.W., Green L.E. (2008). Associations between sole ulcer, white line disease and digital dermatitis and the milk yield of 1,824 dairy cows on 30 dairy cow farms in England and Wales from February 2003–November 2004. Prev. Vet. Med..

[bib6] Amory J.R., Kloosterman P., Barker Z.E., Wright J.L., Blowey R.W., Green L.E. (2006). Risk factors for reduced locomotion in dairy cattle on nineteen farms in the Netherlands. J. Dairy Sci..

[bib7] Atala N., Akcay E. (2001). Turkiye genelinde sgr paratuberkulozu prevalansnn ELISA ile arastrlmas. Etlik Vet. Mikrobiyol. Derg..

[bib8] Atashi H., Abdolmohammadi A., Dadpasand M., Asaadi A. (2012). Prevalence, risk factors and consequent effect of dystocia in Holstein dairy cows in Iran. Asian-Australas. J. Anim. Sci..

[bib9] Atashi H., Abdolmohammadi A.R., Asaadi A., Akhlaghi A., Dadpasand M., Jafari Ahangari Y. (2012). Using an incomplete gamma function to quantify the effect of dystocia on the lactation performance of Holstein dairy cows in Iran. J. Dairy Sci..

[bib10] Bache S.M., Wickham H. (2022). magrittr: A forward-pipe operator for R. v. 2.0.3. https://cran.r-project.org/web/packages/magrittr/magrittr.pdf.

[bib11] Bagri D., Pandey R., Bagri G., Kumari R., Khatik S., Khatik P., Bagdi D. (2018). Impact of subclinical mastitis on milk production in lactating cows in Varanasi. J. Entomol. Zool. Stud..

[bib12] Balduzzi S., Rücker G., Schwarzer G. (2019). How to perform a meta-analysis with R: a practical tutorial. Evid. Based Ment. Health.

[bib13] Bareille N., Beaudeau F., Billon S., Robert A., Faverdin P. (2003). Effects of health disorders on feed intake and milk production in dairy cows. Livest. Prod. Sci..

[bib14] Barker Z.E., Leach K.A., Whay H.R., Bell N.J., Main D.C.J. (2010). Assessment of lameness prevalence and associated risk factors in dairy herds in England and Wales. J. Dairy Sci..

[bib15] Barrier A.C., Haskell M.J. (2011). Calving difficulty in dairy cows has a longer effect on saleable milk yield than on estimated milk production. J. Dairy Sci..

[bib16] Bates T.W., Thurmond M.C., Carpenter T.E. (2003). Description of an epidemic simulation model for use in evaluating strategies to control an outbreak of foot-and-mouth disease. Am. J. Vet. Res..

[bib17] Bauman C.A., Barkema H., Dubuc J., Keefe G., Kelton D. (2016). Identifying management and disease priorities of Canadian dairy industry stakeholders. J. Dairy Sci..

[bib18] Beaudeau F., Ducrocq V., Fourichon C., Seegers H. (1995). Effect of disease on length of productive life of French Holstein dairy cows assessed by survival analysis. J. Dairy Sci..

[bib19] Bellows D.S., Ott S.L., Bellows R.A. (2002). Review: Cost of reproductive diseases and conditions in cattle. Prof. Anim. Sci..

[bib20] Bennett R.M., Christiansen K., Clifton-Hadley R.S. (1999). Estimating the costs associated with endemic diseases of dairy cattle. J. Dairy Res..

[bib21] Beyi A. (2012).

[bib22] Bigras-Poulin M., Meek A.H., Martin S.W. (1990). Interrelationships of health problems and age on milk production in selected Ontario Holstein cows. Prev. Vet. Med..

[bib23] Bonfatti V., Ho P.N., Pryce J.E. (2020). Usefulness of milk mid-infrared spectroscopy for predicting lameness score in dairy cows. J. Dairy Sci..

[bib24] Booth C.J., Warnick L.D., Gröhn Y.T., Maizon D.O., Guard C.L., Janssen D. (2004). Effect of lameness on culling in dairy cows. J. Dairy Sci..

[bib25] Borş S.-I., Borş A. (2020). Ovarian cysts, an anovulatory condition in dairy cattle. J. Vet. Med. Sci..

[bib26] Borsberry S., Dobson H. (1989). Periparturient diseases and their effect on reproductive performance in five dairy herds. Vet. Rec..

[bib27] Boujenane I., El Aimani J., By K. (2015). Effects of clinical mastitis on reproductive and milk performance of Holstein cows in Morocco. Trop. Anim. Health Prod..

[bib28] Bran J.A., Daros R.R., von Keyserlingk M.A.G., LeBlanc S.J., Hötzel M.J. (2018). Cow- and herd-level factors associated with lameness in small-scale grazing dairy herds in Brazil. Prev. Vet. Med..

[bib29] Branscum A.J., Gardner I.A., Johnson W.O. (2005). Estimation of diagnostic-test sensitivity and specificity through Bayesian modeling. Prev. Vet. Med..

[bib30] Branscum A.J., Johnson W.O., Thurmond M.C. (2007). Bayesian beta regression: Applications to household expenditure data and genetic distance between foot-and-mouth disease viruses. Aust. N. Z. J. Stat..

[bib31] Browne N., Hudson C.D., Crossley R.E., Sugrue K., Kennedy E., Huxley J.N., Conneely M. (2022). Cow- and herd-level risk factors for lameness in partly housed pasture-based dairy cows. J. Dairy Sci..

[bib32] Caixeta L.S., Herman J.A., Johnson G.W., McArt J.A.A. (2018). Herd-level monitoring and prevention of displaced abomasum in dairy cattle. Vet. Clin. North Am. Food Anim. Pract..

[bib33] Campos C.C., do Prado F.L., dos Reis J.P.J., Carneiro L.C., Silva P.R.B., de Moraes G.F., dos Santos R.M. (2020). Effects of clinical mastitis and puerperal diseases on reproductive efficiency of dairy cows. Trop. Anim. Health Prod..

[bib34] Cattaneo L., Signorini M., Bertoli J., Bartolomé J., Gareis N., Díaz P., Bó G., Ortega H. (2014). Epidemiological description of cystic ovarian disease in Argentine dairy herds: Risk Factors and effects on the reproductive performance of lactating cows. Reprod. Domest. Anim..

[bib35] Chapinal N., Liang Y., Weary D.M., Wang Y., von Keyserlingk M.A.G. (2014). Risk factors for lameness and hock injuries in Holstein herds in China. J. Dairy Sci..

[bib36] Chapinal N., von Keyserlingk M.A.G., Cerri R.L.A., Ito K., LeBlanc S.J., Weary D.M. (2013). Short communication: Herd-level reproductive performance and its relationship with lameness and leg injuries in freestall dairy herds in the northeastern United States. J. Dairy Sci..

[bib37] Clarkson M.J., Downham D.Y., Faull W.B., Hughes J.W., Manson F.J., Merritt J.B., Murray R.D., Russell W.B., Sutherst J.E., Ward W.R. (1996). Incidence and prevalence of lameness in dairy cattle. Vet. Rec..

[bib38] Costa J.H.C., Burnett T.A., von Keyserlingk M.A.G., Hötzel M.J. (2018). Prevalence of lameness and leg lesions of lactating dairy cows housed in southern Brazil: Effects of housing systems. J. Dairy Sci..

[bib39] Cruz I., Pereira I., Ruprechter G., Barca J., Meikle A., Larriestra A. (2021). Clinical disease incidence during early lactation, risk factors and association with fertility and culling in grazing dairy cows in Uruguay. Prev. Vet. Med..

[bib40] Dawod A., Min B.R. (2014). Effect of puerperal metritis on Holstein cows productive, reproductive variables and culling rates. Am. J. Anim. Vet. Sci..

[bib41] DeGaris P.J., Lean I.J. (2008). Milk fever in dairy cows: A review of pathophysiology and control principles. Vet. J..

[bib42] Dematawewa C.M.B., Berger P.J. (1997). Effect of dystocia on yield, fertility, and cow losses and an economic evaluation of dystocia scores for Holsteins. J. Dairy Sci..

[bib43] Dembele I., Špinka M., Stěhulová I., Panamá J., Firla P. (2006). Factors contributing to the incidence and prevalence of lameness on Czech dairy farms. Czech J. Anim. Sci..

[bib44] Denis-Robichaud J., Kelton D., Fauteux V., Villettaz-Robichaud M., Dubuc J. (2020). Short communication: Accuracy of estimation of lameness, injury, and cleanliness prevalence by dairy farmers and veterinarians. J. Dairy Sci..

[bib45] Dervishi E., Ametaj B.N., Ametaj B.N. (2017). Periparturient Diseases of Dairy Cows: A Systems Biology Approach.

[bib46] Detilleux J.C., Gröhn Y., Eicker S., Quaas R. (1997). Effects of left displaced abomasum on test day milk yields of Holstein cows. J. Dairy Sci..

[bib47] Devleesschauwer B., Torgerson P., Charlier J., Levecke B., Praet N., Roelandt S., Smit S., Dorny P., Berkvens D., Speybroeck N. (2022). prevalence: Tools for prevalence assessment studies. v. 0.4.1. https://rdrr.io/cran/prevalence/.

[bib48] Dijkhuizen A.A., Morris R.S. (1997).

[bib49] Dippel S., Dolezal M., Brenninkmeyer C., Brinkmann J., March S., Knierim U., Winckler C. (2009). Risk factors for lameness in freestall-housed dairy cows across two breeds, farming systems, and countries. J. Dairy Sci..

[bib50] Djemali M., Freeman A.E., Berger P.J. (1987). Reporting of dystocia scores and effects of dystocia on production, days open, and days dry from dairy herd improvement data. J. Dairy Sci..

[bib51] Dohoo I.R., Martin S.W. (1984). Disease, production and culling in Holstein-Friesian cows III. Disease and production as determinants of disease. Prev. Vet. Med..

[bib52] Dolecheck K., Bewley J. (2018). Animal board invited review: Dairy cow lameness expenditures, losses and total cost. Animal.

[bib53] Donat K., Eulenberger K., Kampfer P. (2005). Blutserologische Untersuchungen zur Verbreitung von *Mycobacterium avium* spp. *paratuberculosis* in sachsischen Rinderbestanden. Tierarztl. Umsch..

[bib54] Dubey J.P., Schares G., Ortega-Mora L. (2007). Epidemiology and control of neosporosis and *Neospora caninum*. Clin. Microbiol. Rev..

[bib55] Dubuc J., Duffield T.F., Leslie K.E., Walton J.S., LeBlanc S.J. (2011). Effects of postpartum uterine diseases on milk production and culling in dairy cows. J. Dairy Sci..

[bib56] Duffield T. (2000). Subclinical ketosis in lactating dairy cattle. Vet. Clin. North Am. Food Anim. Pract..

[bib57] Duffield T. (2022).

[bib58] Duffield T.F., Sandals D., Leslie K.E., Lissemore K., McBride B.W., Lumsden J.H., Dick P., Bagg R. (1998). Efficacy of monensin for the prevention of subclinical ketosis in lactating dairy cows. J. Dairy Sci..

[bib59] Dunnington D. (2023). ggspatial: Spatial data framework for ggplot2. v. 1.1.8. https://cran.r-project.org/web/packages/ggspatial/index.html.

[bib60] Eppe J., Lowie T., Opsomer G., Hanley-Cook G., Meesters M., Bossaert P. (2021). Treatment protocols and management of retained fetal membranes in cattle by rural practitioners in Belgium. Prev. Vet. Med..

[bib61] Erb H.N., Martin S.W., Ison N., Swaminathan S. (1981). Interrelationships between production and reproductive diseases in Holstein Cows. Conditional relationships between production and disease. J. Dairy Sci..

[bib62] Erb H.N., Martin S.W., Ison N., Swaminathan S. (1981). interrelationships between production and reproductive diseases in Holstein Cows. Path analysis. J. Dairy Sci..

[bib63] Erb H.N., Smith R.D., Oltenacu P.A., Guard C.L., Hillman R.B., Powers P.A., Smith M.C., White M.E. (1985). Path model of reproductive disorders and performance, milk fever, mastitis, milk yield, and culling in Holstein cows. J. Dairy Sci..

[bib64] Erskine R. (2022).

[bib65] Espejo L.A., Endres M.I., Salfer J.A. (2006). Prevalence of lameness in high-producing Holstein cows housed in freestall barns in Minnesota. J. Dairy Sci..

[bib66] FAOSTAT (2023). https://www.fao.org/faostat/en/#data/OA.

[bib67] FAOSTAT (2023). https://www.fao.org/faostat/en/#data/QCL.

[bib68] FAOSTAT (2023). https://www.fao.org/faostat/en/#data/PP.

[bib69] Fecteau M.E., Whitlock R.H., Behr M.A., Collins D.M. (2010). Paratuberculosis: Organism, Disease, Control.

[bib70] Fernandes L., Guimaraes I., Noyes N., Caixeta L., Machado V. (2021). Effect of subclinical mastitis detected in the first month of lactation on somatic cell count linear scores, milk yield, fertility, and culling of dairy cows in certified organic herds. J. Dairy Sci..

[bib71] Fiore F., Musina D., Cocco R., Di Cerbo A., Spissu N. (2018). Association between left-displaced abomasum corrected with 2-step laparoscopic abomasopexy and milk production in a commercial dairy farm in Italy. Ir. Vet. J..

[bib72] Fourichon C., Beaudeau F., Bareille N., Seegers H. (2001). Incidence of health disorders in dairy farming systems in western France. Livest. Prod. Sci..

[bib73] Fourichon C., Seegers H., Bareille N., Beaudeau F. (1999). Effects of disease on milk production in the dairy cow: A review. Prev. Vet. Med..

[bib74] Fourichon C., Seegers H., Malher X. (2000). Effect of disease on reproduction in the dairy cow: A meta-analysis. Theriogenology.

[bib75] Fukushima Y., Kino E., Furutani A., Minamino T., Honkawa K., Horii Y., Sasaki Y. (2022). Effect of major diseases on productivity of a large dairy farm in a temperate zone in Japan. Dairy.

[bib76] Gaafar H.M.A., Shamiah S.M., El-Hamd M.A.A., Shitta A.A., El-Din M.A.T. (2011). Dystocia in Friesian cows and its effects on postpartum reproductive performance and milk production. Trop. Anim. Health Prod..

[bib77] Garcia S.N., Osburn B.I., Cullor J.S. (2019). A one health perspective on dairy production and dairy food safety. One Health.

[bib78] Garverick H.A. (1997). Ovarian follicular cysts in dairy cows. J. Dairy Sci..

[bib79] GBD (Global Burden of Disease) (2023). https://www.iapb.org/learn/vision-atlas/about/definitions-and-regions/.

[bib80] Geishauser T., Shoukri M., Kelton D., Leslie K. (1998). Analysis of survivorship after displaced abomasum is diagnosed in dairy cows. J. Dairy Sci..

[bib81] Ghavi Hossein-Zadeh N. (2016). Effect of dystocia on subsequent reproductive performance and functional longevity in Holstein cows. J. Anim. Physiol. Anim. Nutr. (Berl.).

[bib82] Giuliodori M.J., Magnasco R.P., Becu-Villalobos D., Lacau-Mengido I.M., Risco C.A., de la Sota R.L. (2013). Metritis in dairy cows: Risk factors and reproductive performance. J. Dairy Sci..

[bib83] Goff J.P. (2008). The monitoring, prevention, and treatment of milk fever and subclinical hypocalcemia in dairy cows. Vet. J..

[bib84] Gorbach S.L. (2000). Probiotics and gastrointestinal health. Am. J. Gastroenterol..

[bib85] Green L.E., Hedges V.J., Schukken Y.H., Blowey R.W., Packington A.J. (2002). The impact of clinical lameness on the milk yield of dairy cows. J. Dairy Sci..

[bib86] Green M.J., Green L.E., Schukken Y.H., Bradley A.J., Peeler E.J., Barkema H.W., de Haas Y., Collis V.J., Medley G.F. (2004). Somatic cell count distributions during lactation predict clinical mastitis. J. Dairy Sci..

[bib87] Griffiths B.E., Grove White D., Oikonomou G. (2018). A cross-sectional study into the prevalence of dairy cattle lameness and associated herd-level risk factors in England and Wales. Front. Vet. Sci..

[bib88] Gröhn Y., Eicker S., Ducrocq V., Hertl J. (1998). Effect of diseases on the culling of Holstein dairy cows in New York State. J. Dairy Sci..

[bib89] Gröhn Y.T., Erb H.N., McCulloch C.E., Saloniemi H.S. (1990). Epidemiology of reproductive disorders in dairy cattle: Associations among host characteristics, disease and production. Prev. Vet. Med..

[bib90] Gröhn Y.T., Eicker S.W., Hertl J.A. (1995). The association between previous 305-day milk yield and disease in New York State dairy cows. J. Dairy Sci..

[bib91] Gröhn Y.T., Erb H.N., McCulloch C.E., Saloniemi H.S. (1989). Epidemiology of metabolic disorders in dairy cattle: Association among host characteristics, disease, and production. J. Dairy Sci..

[bib92] Gu Z., Gu L., Eils R., Schlesner M., Brors B. (2014). circlize Implements and enhances circular visualization in R. Bioinformatics.

[bib93] Gunay A., Gunay U. (2008). Effects of clinical mastitis on reproductive performance in Holstein cows. Acta Vet. Brno.

[bib94] Haine D., Delgado H., Cue R., Sewalem A., Wade K., Lacroix R., Lefebvre D., Arsenault J., Bouchard É., Dubuc J. (2017). Marginal structural Cox model to estimate the causal effect of clinical mastitis on Québec dairy cow culling risk. Prev. Vet. Med..

[bib95] Harrell F. (2023). Hmisc: Harrell miscellaneous. R package version 5.0-1. https://mirrors.nics.utk.edu/cran/web/packages/Hmisc/Hmisc.pdf.

[bib96] Häsler B., Regula G., Stärk K.D.C., Sager H., Gottstein B., Reist M. (2006). Financial analysis of various strategies for the control of Neospora caninum in dairy cattle in Switzerland. Prev. Vet. Med..

[bib97] Hayes E.P.B., Christley R.M., Dobson H. (2012). Effects of periparturient events on subsequent culling and fertility in eight UK dairy herds. Vet. Rec..

[bib98] Heikkilä A.-M., Liski E., Pyörälä S., Taponen S. (2018). Pathogen-specific production losses in bovine mastitis. J. Dairy Sci..

[bib99] Hendrick S.H., Kelton D.F., Leslie K.E., Lissemore K.D., Archambault M., Duffield T.F. (2005). Effect of paratuberculosis on culling, milk production, and milk quality in dairy herds. J. Am. Vet. Med. Assoc..

[bib100] Hernandez J., Shearer J.K., Webb D.W. (2002). Effect of lameness on milk yield in dairy cows. J. Am. Vet. Med. Assoc..

[bib101] Hertl J.A., Schukken Y., Bar D., Bennett G., González R., Rauch B., Welcome F., Tauer L., Gröhn Y. (2011). The effect of recurrent episodes of clinical mastitis caused by gram-positive and gram-negative bacteria and other organisms on mortality and culling in Holstein dairy cows. J. Dairy Sci..

[bib102] Hertl J.A., Schukken Y.H., Tauer L.W., Welcome F.L., Gröhn Y.T. (2018). Does clinical mastitis in the first 100 days of lactation 1 predict increased mastitis occurrence and shorter herd life in dairy cows?. J. Dairy Sci..

[bib103] Hijmans R., Barbosa M., Ghosh A., Mandel A. (2023). geodata: Download geographic data. v. 0.5-9. https://cran.r-project.org/web/packages/geodata/index.html.

[bib104] Horst H.S., Dijkhuizen A.A., Huirne R.B.M., Meuwissen M.P.M. (1999). Monte Carlo simulation of virus introduction into the Netherlands. Prev. Vet. Med..

[bib105] Horst R.L., Goff J.P., Reinhardt T.A., Buxton D.R. (1997). Strategies for preventing milk fever in dairy cattle. J. Dairy Sci..

[bib106] Hossein-Zadeh N.G. (2014). Effect of dystocia on the productive performance and calf stillbirth in Iranian Holsteins. J. Agric. Sci. Technol..

[bib107] Huntington B., Bernardo T.M., Bondad-Reantaso M., Bruce M., Devleesschauwer B., Gilbert W., Grace D., Havelaar A., Herrero M., Marsh T.L., Mesenhowski S., Pendell D., Pigott D., Shaw A.P., Stacey D., Stone M., Torgerson P., Watkins K., Weiland B., Rushton J. (2021). Global Burden of Animal Diseases: A novel approach to understanding and managing disease in livestock and aquaculture. Rev. Sci. Tech..

[bib108] Hvitfeldt E. (2021). paletteer: Comprehensive collection of color palettes. v. 1.3.0. https://cran.r-project.org/web/packages/paletteer/index.html.

[bib109] IFCN (2023). IFCN Methods - 3. IFCN Typical Farm Approach. IFCN AG, the Dairy Research Network. https://ifcndairy.org/about-us/ifcn-dairy-research-network-method/.

[bib110] Jensen K.C., Oehm A.W., Campe A., Stock A., Woudstra S., Feist M., Müller K.E., Hoedemaker M., Merle R. (2022). German farmers' awareness of lameness in their dairy herds. Front. Vet. Sci..

[bib111] Jewell M.T., Cameron M., Spears J., McKenna S.L., Cockram M.S., Sanchez J., Keefe G.P. (2019). Prevalence of lameness and associated risk factors on dairy farms in the Maritime Provinces of Canada. J. Dairy Sci..

[bib112] Johnson-Ifearulundu Y.J., Kaneene J.B., Sprecher D.J., Gardiner J.C., Lloyd J.W. (2000). The effect of subclinical *Mycobacterium paratuberculosis* infection on days open in Michigan, USA, dairy cows. Prev. Vet. Med..

[bib113] Jorgensen N.A. (1974). Combating milk fever. J. Dairy Sci..

[bib114] Jorritsma R., Westerlaan B., Bierma M.P.R., Frankena K. (2008). Milk yield and survival of Holstein-Friesian dairy cattle after laparoscopic correction of left-displaced abomasum. Vet. Rec..

[bib115] Jurkovich V., Bognár B., Balogh K., Kovács-Weber M., Fornyos K., Szabó R.T., Kovács P., Könyves L., Mézes M. (2016). Effects of subclinical *Mycobacterium avium* ssp. *paratuberculosis* infection on some physiological parameters, health status and production in dairy cows. Acta Vet. Hung..

[bib116] Kamel E.R., Ahmed H.A., Hassan F.M. (2022). The effect of retained placenta on the reproductive performance and its economic losses in a Holstein dairy herd. Iraqi J. Vet. Sci..

[bib117] Kaneene J.B., Miller R. (1995). Risk factors for metritis in Michigan dairy cattle using herd- and cow-based modelling approaches. Prev. Vet. Med..

[bib118] Karsten S., Rave G., Krieter J. (2005). Monte Carlo simulation of classical swine fever epidemics and control: I. General concepts and description of the model. Vet. Microbiol..

[bib119] Kesler D.J., Garverick H.A. (1982). Ovarian cysts in dairy cattle: A review. J. Anim. Sci..

[bib120] King M.T.M., LeBlanc S.J., Pajor E.A., DeVries T.J. (2017). Cow-level associations of lameness, behavior, and milk yield of cows milked in automated systems. J. Dairy Sci..

[bib121] King M.T.M., Pajor E.A., LeBlanc S.J., DeVries T.J. (2016). Associations of herd-level housing, management, and lameness prevalence with productivity and cow behavior in herds with automated milking systems. J. Dairy Sci..

[bib122] Klaas I.C., Wessels U., Rothfuss H., Tenhagen B.A., Heuwieser W., Schallenberger E. (2004). Factors affecting reproductive performance in German Holstein-Friesian cows with a special focus on postpartum mastitis. Livest. Prod. Sci..

[bib123] Kocak O., Ekiz B. (2006). The effect of lameness on milk yield in dairy cows. Acta Vet. Brno.

[bib124] Könyves L., Szenci O., Jurkovich V., Tegzes L., Tirián A., Solymosi N., Gyulay G., Brydl E. (2009). Risk assessment and consequences of retained placenta for uterine health, reproduction and milk yield in dairy cows. Acta Vet. Brno.

[bib125] Kossaibati M.A., Hovi M., Esslemont R.J. (1998). Incidence of clinical mastitis in dairy herds in England. Vet. Rec..

[bib126] Krishnamoorthy P., Goudar A.L., Suresh K.P., Roy P. (2021). Global and countrywide prevalence of subclinical and clinical mastitis in dairy cattle and buffaloes by systematic review and meta-analysis. Res. Vet. Sci..

[bib127] Kudahl A.B., Østergaard S., Sørensen J.T., Nielsen S.S. (2007). A stochastic model simulating paratuberculosis in a dairy herd. Prev. Vet. Med..

[bib128] Kumari S., Kumaresan A., Patbandha T.K., Ravi S.K. (2016). Risk factors for metritis and its effect on productive and reproductive performance in dairy cattle and buffaloes. Agric. Res..

[bib129] Kumari S., Prasad S., Kumaresan A., Manimaran A., Patbandha T.K., Pathak R., Boro P., Mohanty T.K., Ravi S.K. (2015). Risk factors and impact of retained fetal membranes on performance of dairy bovines reared under subtropical conditions. Trop. Anim. Health Prod..

[bib130] Laven R.A., Peters A. (1996). Bovine retained placenta: Aetiology, pathogenesis and economic loss. Vet. Rec..

[bib131] Leach K.A., Whay H., Maggs C., Barker Z., Paul E., Bell A., Main D. (2010). Working towards a reduction in cattle lameness: 1. Understanding barriers to lameness control on dairy farms. Res. Vet. Sci..

[bib132] LeBlanc S.J., Leslie K.E., Duffield T.F. (2005). Metabolic predictors of displaced abomasum in dairy cattle. J. Dairy Sci..

[bib133] Lima F. (2022).

[bib134] Loiklung C., Sukon P., Thamrongyoswittayakul C. (2022). Global prevalence of subclinical ketosis in dairy cows: A systematic review and meta-analysis. Res. Vet. Sci..

[bib135] Lombard J.E., Garry F.B., McCluskey B.J., Wagner B.A. (2005). Risk of removal and effects on milk production associated with paratuberculosis status in dairy cows. J. Am. Vet. Med. Assoc..

[bib136] López de Maturana E., Ugarte E., González-Recio O. (2007). Impact of Calving Ease on Functional Longevity and Herd Amortization Costs in Basque Holsteins Using Survival Analysis. J. Dairy Sci..

[bib137] Mahnani A., Sadeghi-Sefidmazgi A., Ansari-Mahyari S., Ghorbani G.-R. (2021). Assessing the consequences and economic impact of retained placenta in Holstein dairy cattle. Theriogenology.

[bib138] Mahnani A., Sadeghi-Sefidmazgi A., Cabrera V.E. (2015). Consequences and economics of metritis in Iranian Holstein dairy farms. J. Dairy Sci..

[bib139] Malašauskienė D., Antanaitis R., Juozaitienė V., Paulauskas A., Urbonavičius G., Televičius M., Urbutis M., Kajokienė L., Yilmaz A., Baumgartner W. (2022). Impact of calving difficulty on lameness in dairy cows. Agriculture.

[bib140] Mangurkar B.R., Hayes J.F., Moxley J.E. (1984). Effects of calving ease-calf survival on production and reproduction in Holsteins. J. Dairy Sci..

[bib141] Manske T., Hultgren J., Bergsten C. (2002). Prevalence and interrelationships of hoof lesions and lameness in Swedish dairy cows. Prev. Vet. Med..

[bib142] Martins L., Barcelos M.M., Cue R.I., Anderson K.L., Santos M.V., Gonçalves J.L. (2020). Chronic subclinical mastitis reduces milk and components yield at the cow level. J. Dairy Res..

[bib143] Matson R.D., King M.T.M., Duffield T.F., Santschi D.E., Orsel K., Pajor E.A., Penner G.B., Mutsvangwa T., DeVries T.J. (2022). Farm-level factors associated with lameness prevalence, productivity, and milk quality in farms with automated milking systems. J. Dairy Sci..

[bib144] McAloon C.G., Whyte P., More S.J., Green M.J., O'Grady L., Garcia A., Doherty M.L. (2016). The effect of paratuberculosis on milk yield—A systematic review and meta-analysis. J. Dairy Sci..

[bib145] McCabe L.D., Martin B.R., McCabe G.P., Johnston C.C., Weaver C.M., Peacock M. (2004). Dairy intakes affect bone density in the elderly. Am. J. Clin. Nutr..

[bib146] McDonald S.A., Haagsma J.A., Cassini A., Devleesschauwer B. (2020). Adjusting for comorbidity in incidence-based DALY calculations: An individual-based modeling approach. BMC Med. Res. Methodol..

[bib147] Melendez P., Bartolome J., Archbald L.F., Donovan A. (2003). The association between lameness, ovarian cysts and fertility in lactating dairy cows. Theriogenology.

[bib148] Mellado M., García J.E., Véliz Deras F.G., de Santiago M.Á., Mellado J., Gaytán L.R., Ángel-García O. (2018). The effects of periparturient events, mastitis, lameness and ketosis on reproductive performance of Holstein cows in a hot environment. Austral J. Vet. Sci..

[bib149] Moreira T.F., Nicolino R.R., de Andrade L.S., Filho E.J.F., de Carvalho A.U. (2018). Prevalence of lameness and hoof lesions in all year-round grazing cattle in Brazil. Trop. Anim. Health Prod..

[bib150] Mostert P.F., Bokkers E.A.M., van Middelaar C.E., Hogeveen H., de Boer I.J.M. (2018). Estimating the economic impact of subclinical ketosis in dairy cattle using a dynamic stochastic simulation model. Animal.

[bib151] Mujuni P.F., Mgongo F.O.K., Kanuya N.L. (1993). Ovarian cysts, a postpartum ovarian disorder affecting dairy cows in a tropical area. Anim. Reprod. Sci..

[bib152] Nielsen S.S., Toft N. (2009). A review of prevalences of paratuberculosis in farmed animals in Europe. Prev. Vet. Med..

[bib153] Oetzel G. (2013).

[bib154] Oetzel G.R. (2004). Monitoring and testing dairy herds for metabolic disease. Vet. Clin. North Am. Food Anim. Pract..

[bib155] Olesen J., Gustavsson A., Svensson M., Wittchen H.U., Jönsson B., CDBE2010 Study Group, European Brain Council (2012). The economic cost of brain disorders in Europe. Eur. J. Neurol..

[bib156] Ooms J. (2023). writexl: Export data frames to Excel 'xlsx' format. v. 1.4.2. https://cran.r-project.org/web/packages/writexl/writexl.pdf.

[bib157] Østergaard S., Ettema J.F., Hjortø L., Pedersen J., Lassen J., Kargo M. (2016). Avoiding double counting when deriving economic values through stochastic dairy herd simulation. Livest. Sci..

[bib158] Ott S.L., Wells S.J., Wagner B.A. (1999). Herd-level economic losses associated with Johne's disease on US dairy operations. Prev. Vet. Med..

[bib159] Ózsvári L. (2017). Economic cost of lameness in dairy cattle herds. J. Dairy Vet. Anim. Res.

[bib160] Ózsvári L., Barna R., Visnyei L. (2007). Economic losses due to bovine foot diseases in large-scale Holstein-Friesian dairy herds. Magy. Állatorv. Lapja.

[bib161] Ózsvári L., Harnos A., Lang Z., Monostori A., Strain S., Fodor I. (2020). The impact of paratuberculosis on milk production, fertility, and culling in large commercial Hungarian dairy herds. Front. Vet. Sci..

[bib162] Paule R.C., Mandel J. (1982). Consensus values and weighting factors. J. Res. Natl. Bur. Stand..

[bib163] Peake K.A., Biggs A.M., Argo C.M., Smith R.F., Christley R.M., Routly J.E., Dobson H. (2011). Effects of lameness, subclinical mastitis and loss of body condition on the reproductive performance of dairy cows. Vet. Rec..

[bib164] Pebesma E. (2018). Simple features for R: Standardized support for spatial vector data. R J..

[bib165] Peterson C.B., Mitloehner F.M. (2021). Sustainability of the dairy industry: Emissions and mitigation opportunities. Front. Anim. Sci..

[bib166] Petit E. (2001). Enquête sérologique sur la paratuberculose bovine menée dans l'Yonne lors de la campagne 98–99. Epidémiol. Santé Anim..

[bib167] Pfützner M., Ózsvari L. (2017). The financial impact of decreased milk production due to subclinical mastitis in German dairy herds. Istanbul Univ. Vet. Fak. Derg..

[bib168] Piccardi M., Romero G., Veneranda G., Castello E., Romero D., Balzarini M., Bó G.A. (2016). Effect of puerperal metritis on reproductive and productive performance in dairy cows in Argentina. Theriogenology.

[bib169] Plummer M., Stukalov A., Denwood M. (2023). Bayesian graphical models using MCMC. v. 4-15. https://cran.r-project.org/web/packages/rjags/rjags.pdf.

[bib170] Pouillot R., Delignette-Muller M.L. (2010). Evaluating variability and uncertainty separately in microbial quantitative risk assessment using two R packages. Int. J. Food Microbiol..

[bib171] Prasomsri P. (2022). Effect of lameness on daily milk yield in dairy cow. Wetchasan Sattawaphaet.

[bib172] Probo M., Pascottini O.B., LeBlanc S., Opsomer G., Hostens M. (2018). Association between metabolic diseases and the culling risk of high-yielding dairy cows in a transition management facility using survival and decision tree analysis. J. Dairy Sci..

[bib173] Puerto M.A., Shepley E., Cue R.I., Warner D., Dubuc J., Vasseur E. (2021). The hidden cost of disease: II. Impact of the first incidence of lameness on production and economic indicators of primiparous dairy cows. J. Dairy Sci..

[bib174] R Core Team (2023).

[bib175] Raboisson D., Mounié M., Khenifar E., Maigné E. (2015). The economic impact of subclinical ketosis at the farm level: Tackling the challenge of over-estimation due to multiple interactions. Prev. Vet. Med..

[bib176] Raboisson D., Mounié M., Maigné E. (2014). Diseases, reproductive performance, and changes in milk production associated with subclinical ketosis in dairy cows: A meta-analysis and review. J. Dairy Sci..

[bib177] Raizman E.A., Santos J. (2002). The effect of left displacement of abomasum corrected by toggle-pin suture on lactation, reproduction, and health of Holstein dairy cows. J. Dairy Sci..

[bib178] Raizman E.A., Fetrow J.P., Wells S.J. (2009). Loss of income from cows shedding *Mycobacterium avium* subspecies *paratuberculosis* prior to calving compared with cows not shedding the organism on two Minnesota dairy farms. J. Dairy Sci..

[bib179] Rajala P.J., Gröhn Y.T. (1998). Effects of dystocia, retained placenta, and metritis on milk yield in dairy cows. J. Dairy Sci..

[bib180] Rajala-Schultz P.J., Gröhn Y.T. (1999). Culling of dairy cows. Part I. Effects of diseases on culling in Finnish Ayrshire cows. Prev. Vet. Med..

[bib181] Ramos O.P., Rezende A.L., de Alvarenga P.B., Campos C.C., de Rezende E.V., Silva M.J.B., Carneiro L.C., de Moraes G.F., Saut J.P.E., dos Santos R.M. (2022). Effect of retained placenta and clinical mastitis on reproduction parameters, immune response, and steroidogenic receptors gene expression in postpartum crossbred dairy cows. Trop. Anim. Health Prod..

[bib182] Randall L.V., Thomas H.J., Remnant J.G., Bollard N.J., Huxley J.N. (2019). Lameness prevalence in a random sample of UK dairy herds. Vet. Rec..

[bib183] Ranjbar S., Rabiee A.R., Ingenhoff L., House J.K. (2020). Farmers' perceptions and approaches to detection, treatment and prevention of lameness in pasture-based dairy herds in New South Wales, Australia. Aust. Vet. J..

[bib184] Rasmussen P., Barkema H.W., Beaulieu E., Mason S., Hall D.C. (2021). Estimation of the value of Johne's disease (paratuberculosis) control to Canadian dairy producers. Prev. Vet. Med..

[bib185] Rasmussen P., Barkema H.W., Beaulieu E., Mason S., Hall D.C. (2022). Economic premiums associated with *Mycobacterium avium* ssp. *paratuberculosis*-negative replacement purchases in major dairy-producing regions. J. Dairy Sci..

[bib186] Rasmussen P., Barkema H.W., Hall D.C. (2021). Effectiveness and economic viability of Johne's disease (paratuberculosis) control practices in dairy herds. Front. Vet. Sci..

[bib187] Rasmussen P., Barkema H.W., Mason S., Beaulieu E., Hall D.C. (2021). Economic losses due to Johne's disease (paratuberculosis) in dairy cattle. J. Dairy Sci..

[bib188] Rasmussen P., Shaw A., Jemberu W., Knight-Jones T., Conrady B., Apenteng O., Cheng Y., Munoz V., Rushton J., Torgerson P. (2024). Economic losses due to foot-and-mouth disease (FMD) in Ethiopian cattle. Prev. Vet. Med..

[bib189] Rasmussen P., Shaw A.P.M., Muñoz V., Bruce M., Torgerson P.R. (2022). Estimating the burden of multiple endemic diseases and health conditions using Bayes' Theorem: A conditional probability model applied to UK dairy cattle. Prev. Vet. Med..

[bib190] Reichel M.P., Alejandra Ayanegui-Alcérreca M., Gondim L.F.P., Ellis J.T. (2013). What is the global economic impact of *Neospora caninum* in cattle—The billion dollar question. Int. J. Parasitol..

[bib191] Reppert E.J. (2015). Evidence for the use of ceftiofur for treatment of metritis in dairy cattle. Vet. Clin. North Am. Food Anim. Pract..

[bib192] Robbi C., Rossi I., Nardelli S., Rossi E., Toson M., Marangon S., Vincenzi G., Vicenzoni G. (2002). Proc. 34th Nat. Congr. SIB, Marina di Ravenna (RA), Italy.

[bib193] Roberts J. (2022).

[bib194] Roche S.M., Ross J.A., Schatz C., Beaugrand K., Zuidhof S., Ralston B., Allan N., Olson M. (2023). Impact of Dystocia on Milk Production, Somatic Cell Count, Reproduction and Culling in Holstein Dairy Cows. Animals (Basel).

[bib195] Rossi G., Grohn Y.T., Schukken Y.H., Smith R.L. (2017). The effect of *Mycobacterium avium* ssp. *paratuberculosis* infection on clinical mastitis occurrence in dairy cows. J. Dairy Sci..

[bib196] Rouha-Mülleder C., Iben C., Wagner E., Laaha G., Troxler J., Waiblinger S. (2009). Relative importance of factors influencing the prevalence of lameness in Austrian cubicle loose-housed dairy cows. Prev. Vet. Med..

[bib197] Rowlands G.J., Lucey S. (1986). Changes in milk yield in dairy cows associated with metabolic and reproductive disease and lameness. Prev. Vet. Med..

[bib198] RStudio Team (2023).

[bib199] Rushton J., Bruce M., Bellet C., Torgerson P., Shaw A., Marsh T., Pigott D., Stone M., Pinto J., Mesenhowski S., Wood P. (2018). Initiation of global burden of animal diseases programme. Lancet.

[bib200] Rutherford K.M.D., Langford F.M., Jack M.C., Sherwood L., Lawrence A.B., Haskell M.J. (2009). Lameness prevalence and risk factors in organic and non-organic dairy herds in the United Kingdom. Vet. J..

[bib201] Sajid A., Avais M., Aneela Zameer D., Ashraf K., Jabeen S., Hameed S., Muhammad A., Jawaria Ali K., Ahmad I. (2021). Prevalence and Associated Risk Factors of Lameness in Cows at Commercial Dairy Herds in Punjab, Pakistan. Pak. J. Zool..

[bib202] Salar S., Çalişici O., Çalişici D., Özen D., Baştan A. (2019). Negative effects of occurrence of clinical mastitis from calving to end of the voluntary waiting period on reproduction in Holstein cows. Turk. J. Vet. Anim. Sci..

[bib203] Sarjokari K., Kaustell K.O., Hurme T., Kivinen T., Peltoniemi O.A.T., Saloniemi H., Rajala-Schultz P.J. (2013). Prevalence and risk factors for lameness in insulated free stall barns in Finland. Livest. Sci..

[bib204] Šárová R., Stěhulová I., Kratinová P., Firla P., Špinka M. (2011). Farm managers underestimate lameness prevalence in Czech dairy herds. Anim. Welf..

[bib205] Schauberger P. (2023). openxlsx: Read, write and edit xlsx files. R package version 4.2.5.2. https://cran.r-project.org/web/packages/openxlsx/openxlsx.pdf.

[bib206] Schrick F.N., Hockett M.E., Saxton A.M., Lewis M.J., Dowlen H.H., Oliver S.P. (2001). Influence of subclinical mastitis during early lactation on reproductive parameters. J. Dairy Sci..

[bib207] Sharifi H., Kostoulas P., Bahonar A., Bokaie S., Vodjgani M., Haghdoost A.A., Karamouzian M., Rahimi Foroushani A., Leontides L. (2013). Effect of health disorders on the hazard of culling on the first or second lactation in Iranian dairy herds. Prev. Vet. Med..

[bib208] Shephard R.W., Williams Sh.H., Beckett S.D. (2016). Farm economic impacts of bovine Johne's disease in endemically infected Australian dairy herds. Aust. Vet. J..

[bib209] Silvia W.J., Hatler T., Nugent A., Laranja da Fonseca L.L. (2002). Ovarian follicular cysts in dairy cows: An abnormality in folliculogenesis. Domest. Anim. Endocrinol..

[bib210] Simerl N.A., Wilcox C.J., Thatcher W.W. (1992). Postpartum performance of dairy heifers freshening at young ages. J. Dairy Sci..

[bib211] Sjöström K., Fall N., Blanco-Penedo I., Duval J.E., Krieger M., Emanuelson U. (2018). Lameness prevalence and risk factors in organic dairy herds in four European countries. Livest. Sci..

[bib212] Smith J., van Winden S. (2019). Risk of lameness in dairy cows with paratuberculosis infection. Animals (Basel).

[bib213] Smith N.W., Fletcher A.J., Hill J.P., McNabb W.C. (2022). Modeling the contribution of milk to global nutrition. Front. Nutr..

[bib214] Statham J. (2023).

[bib215] Steeneveld W., Amuta P., van Soest F.J.S., Jorritsma R., Hogeveen H. (2020). Estimating the combined costs of clinical and subclinical ketosis in dairy cows. PLoS One.

[bib216] Steeneveld W., van Werven T., Barkema H.W., Hogeveen H. (2011). Cow-specific treatment of clinical mastitis: An economic approach. J. Dairy Sci..

[bib217] Steinbock L. (2006).

[bib218] Stevenson J.S., Call E.P. (1988). Reproductive disorders in the periparturient dairy cow. J. Dairy Sci..

[bib219] Suthar V.S., Canelas-Raposo J., Deniz A., Heuwieser W. (2013). Prevalence of subclinical ketosis and relationships with postpartum diseases in European dairy cows. J. Dairy Sci..

[bib220] Tabe Ojong M.P.J., Hauser M., Mausch K. (2022). Does agricultural commercialisation increase asset and livestock accumulation on smallholder farms in Ethiopia?. J. Dev. Stud..

[bib221] Tenhagen B.A., Helmbold A., Heuwieser W. (2007). Effect of various degrees of dystocia in dairy cattle on calf viability, milk production, fertility and culling. J. Vet. Med. A Physiol. Pathol. Clin. Med..

[bib222] Thomsen P.T., Shearer J.K., Houe H. (2023). Prevalence of lameness in dairy cows: A literature review. Vet. J..

[bib223] Tiwari A., VanLeeuwen J.A., Dohoo I.R., Stryhn H., Keefe G.P., Haddad J.P. (2005). Effects of seropositivity for bovine leukemia virus, bovine viral diarrhoea virus, *Mycobacterium avium* subspecies *paratuberculosis*, and *Neospora caninum* on culling in dairy cattle in four Canadian provinces. Vet. Microbiol..

[bib224] Tiwari A., VanLeeuwen J.A., McKenna S.L., Keefe B.P., Barkema H.W. (2006). Johne's disease in Canada: Part I—Clinical symptoms, pathophysiology, diagnosis, and prevalence in dairy herds. Can. Vet. J..

[bib225] Toni F., Vincenti L., Ricci A., Schukken Y.H. (2015). Postpartum uterine diseases and their impacts on conception and days open in dairy herds in Italy. Theriogenology.

[bib226] Torgerson P.R., Shaw A. (2021). A simple metric to capture losses: The concept of an animal health loss envelope. OIE Bull..

[bib227] Tranter W.P., Morris R.S. (1991). A case study of lameness in three dairy herds. N. Z. Vet. J..

[bib228] Urbanek S., Horner J. (2023). Cairo: R graphics device using Cairo graphics library for creating high-quality bitmap (PNG, JPEG, TIFF), vector (PDF, SVG, PostScript) and display (X11 and Win32) output. v 1.6-2. https://cran.r-project.org/web/packages/Cairo/Cairo.pdf.

[bib229] van den Borne B.H.P., Vernooij J.C.M., Lupindu A.M., van Schaik G., Frankena K., Lam T.J.G.M., Nielen M. (2011). Relationship between somatic cell count status and subsequent clinical mastitis in Dutch dairy cows. Prev. Vet. Med..

[bib230] van Dijk M., Morley T., Rau M.L., Saghai Y. (2021). A meta-analysis of projected global food demand and population at risk of hunger for the period 2010–2050. Nat. Food.

[bib231] van Huyssteen M., Barkema H.W., Mason S., Orsel K. (2020). Association between lameness risk assessment and lameness and foot lesion prevalence on dairy farms in Alberta, Canada. J. Dairy Sci..

[bib232] Vanholder T., Opsomer G., De Kruif A. (2006). Aetiology and pathogenesis of cystic ovarian follicles in dairy cattle: a review. Reprod. Nutr. Dev..

[bib233] Vicenzoni G., Filippi L., Dolci P., Corrò M., Job L., Robbi C., Mutinelli F., Marangon S. (1999). Prevalenza del *Mycobacterium paratuberculosis* in bovine macellate in provincia di Verona. Atti della Societa Italiana di Buiatria.

[bib234] Warner D., Vasseur E., Lefebvre D.M., Lacroix R. (2020). A machine learning based decision aid for lameness in dairy herds using farm-based records. Comput. Electron. Agric..

[bib235] Westin R., Vaughan A., de Passillé A.M., DeVries T.J., Pajor E.A., Pellerin D., Siegford J.M., Witaifi A., Vasseur E., Rushen J. (2016). Cow- and farm-level risk factors for lameness on dairy farms with automated milking systems. J. Dairy Sci..

[bib236] Whay H.R., Main D., Green L., Webster A. (2003). Assessment of the welfare of dairy caftle using animal-based measurements: direct observations and investigation of farm records. Vet. Rec..

[bib237] Whay H.R., Shearer J.K. (2017). The impact of lameness on welfare of the dairy. Vet. Clin. North Am. Food Anim. Pract..

[bib238] Wickham H. (2016).

[bib239] Wickham H. (2023). forcats: Tools for working with categorical variables (factors). v. 1.0.0. https://cran.r-project.org/web/packages/forcats/forcats.pdf.

[bib240] Wickham H. (2023). stringr: Simple, consistent wrappers for common string operations. v. 1.5.1. https://search.r-project.org/CRAN/refmans/stringr/html/stringr-package.html.

[bib241] Wickham H., François R., Henry L., Müller K., Vaughan D. (2023). dplyr: A grammar of data manipulation. v. 1.1.4. https://cloud.r-project.org/web/packages/dplyr/dplyr.pdf.

[bib242] Wickham H., Vaughan D., Girlich M. (2023). tidyr: Tidy messy data. v. 1.3.0. https://cran.r-project.org/web/packages/tidyr/index.html.

[bib243] Wittek T. (2022).

[bib244] World Bank (2023). https://data.worldbank.org/indicator/FP.CPI.TOTL.

[bib245] World Bank (2023). https://data.worldbank.org/indicator/PA.NUS.FCRF.

[bib246] Yilmaz Ö.T. (2017). A study of milk support policies in the European Union and in Turkey. European Journal of Interdisciplinary Studies.

[bib247] Youngquist R.S., Threlfall W.R. (2006).

[bib248] Zemel M.B. (2004). Role of calcium and dairy products in energy partitioning and weight management. Am. J. Clin. Nutr..

